# The *Arabidopsis* U1 snRNP regulates mRNA 3′-end processing

**DOI:** 10.1038/s41477-024-01796-8

**Published:** 2024-09-23

**Authors:** Anchilie F. Mangilet, Joachim Weber, Sandra Schüler, Manon Adler, Eneza Yoeli Mjema, Paula Heilmann, Angie Herold, Monique Renneberg, Luise Nagel, Irina Droste-Borel, Samuel Streicher, Thomas Schmutzer, Gregor Rot, Boris Macek, Cornelius Schmidtke, Sascha Laubinger

**Affiliations:** 1 Institute of Biology and Environmental Sciences, University of Oldenburg, Oldenburg, Germany; 2 Institute of Biology, Department of Genetics, Martin Luther University Halle-Wittenberg, Halle (Saale), Germany; 3 Proteome Center, University of Tuebingen, Tuebingen, Germany; 4 Institute of Agricultural and Nutritional Sciences, Martin Luther University Halle-Wittenberg, Halle (Saale), Germany; 5 Institute of Molecular Life Sciences of the University of Zurich and Swiss Institute of Bioinformatics, Zurich, Switzerland; 6 Present Address: Max Planck Institute for Plant Breeding Research (MPIPZ), Cologne, Germany

**Keywords:** Plant molecular biology, Gene expression analysis, Plant genetics

## Abstract

The removal of introns by the spliceosome is a key gene regulatory mechanism in eukaryotes, with the U1 snRNP subunit playing a crucial role in the early stages of splicing. Studies in metazoans show that the U1 snRNP also conducts splicing-independent functions, but the lack of genetic tools and knowledge about U1 snRNP-associated proteins have limited the study of such splicing-independent functions in plants. Here we describe an RNA-centric approach that identified more than 200 proteins associated with the *Arabidopsis* U1 snRNP and revealed a tight link to mRNA cleavage and polyadenylation factors. Interestingly, we found that the U1 snRNP protects mRNAs against premature cleavage and polyadenylation within introns—a mechanism known as telescripting in metazoans—while also influencing alternative polyadenylation site selection in 3′-UTRs. Overall, our work provides a comprehensive view of U1 snRNP interactors and reveals novel functions in regulating mRNA 3′-end processing in *Arabidopsis*, laying the groundwork for understanding non-canonical functions of plant U1 snRNPs.

## Abstract (web summary)

Researchers found that a plant U1 snRNP complex associates with 200 proteins and conducts splicing-independent roles by safeguarding RNAs against premature cleavage and polyadenylation, similar to a process known as telescripting in metazoans.

## Main

In eukaryotes, the spliceosome removes intronic sequences in messenger RNAs and subsequently ligates exons to generate a functional mRNA. Five uridine-rich small nuclear ribonucleoprotein (snRNP) complexes (U1, U2, U4, U5 and U6 snRNPs) build the spliceosome^1^. Each of the snRNPs is composed of a specific small nuclear RNA (snRNA) and protein subunits that are essential for the recognition of splicing signals embedded in the gene sequences^2^. During the splicing process, the snRNPs assemble in an accurate step-by-step manner. The recognition of the 5′ splice sites (5′SS) by the U1 snRNP initiates the splicing process. Cryo-electron microscopy has facilitated a more detailed dissection of the U1 snRNP function, particularly in the early steps of the splicing reaction^3–5^. The core U1 snRNP consists of a 165-nucleotide snRNA that forms four stem-loops, an Sm core ring (Sm-E, G, D3, B, D1, D2 and F) and three U1 core proteins (U1-A, U1-70K and U1-C)^6,7^. Accessory proteins specifically interact with the U1 core snRNP and aid splicing of weak 5′SS^8–11^. In *Arabidopsis*, core and accessory proteins are conserved, and mutants lacking U1 accessory components such as LUC7, PRP39, PRP40 or PRP45 exhibit developmental defects^12–19^. Surprisingly, while a flower-specific RNA interference (RNAi) knockdown of *U1-70K* shows developmental defects, two reports describing mutants for the U1 core components, U1-A and U1-70K, did not find any drastic effects^20–22^. This is in stark contrast to the fact that U1 core components are essential genes in metazoans^23,24^, and it shows that several aspects of the function of the *Arabidopsis* U1 snRNP in plants are not understood and remain to be analysed.

The U1 snRNP is more abundant than other snRNPs and has early been thought to fulfil additional functions aside from splicing^25^. Indeed, the metazoan U1 snRNP affects mRNA length through regulation of 3′-end processing, controls promoter directionality, enhances transcription, increases the speed of RNA polymerase II (RNAPII) and is responsible for retaining long non-coding RNAs in the nucleus^6,26–30^. Probably the best-described non-canonical function of the metazoan U1 snRNP is telescripting, by which the U1 snRNP prevents premature cleavage and polyadenylation in introns, thereby ensuring transcription of full-length RNAs^31^. The telescripting function is specifically important for long genes, which contain long introns and require intact U1 snRNP to complete transcription at canonical cleavage and polyadenylation (CPA) sites^32^. Environmental cues can also modulate telescripting activity and several human diseases can be linked to telescripting^33–35^. Whether telescripting exists in plants, particularly in plants with rather small introns such as *Arabidopsis*, is currently not known. Mechanistically, the metazoan U1 snRNP forms a complex with cleavage and polyadenylation factors (CPAFs) called U1-CPAF, which is distinct from U1 snRNP spliceosomal complexes^36^. The U1-CPAF complex binds nascent RNAs in introns that contain U1 and CPAF binding sites, but the presence of the U1 snRNP in this complex blocks cleavage-stimulatory factors from joining the complex^36^.

While numerous exciting non-canonical functions of metazoan snRNPs are being constantly discovered, comprehensive knowledge about the interactors of plant U1 snRNPs, as well as genetic tools to study the function of U1 snRNP in plants, is still lacking. In this study, we present the *Arabidopsis* U1 snRNP interactome and, in addition, generate genetic resources to investigate the non-canonical functions of the *Arabidopsis* U1 snRNP. Our findings demonstrate that the *Arabidopsis* U1 snRNP plays a splicing-independent role in 3′-end processing, as it features a telescripting function similar to metazoans while also contributing to alternative polyadenylation in 3′ untranslated regions (3′-UTRs), possibly coupled with a general function in RNAPII termination.

## Results

### A compendium of *Arabidopsis* U1 snRNP-associated proteins

**Fig. 1:**
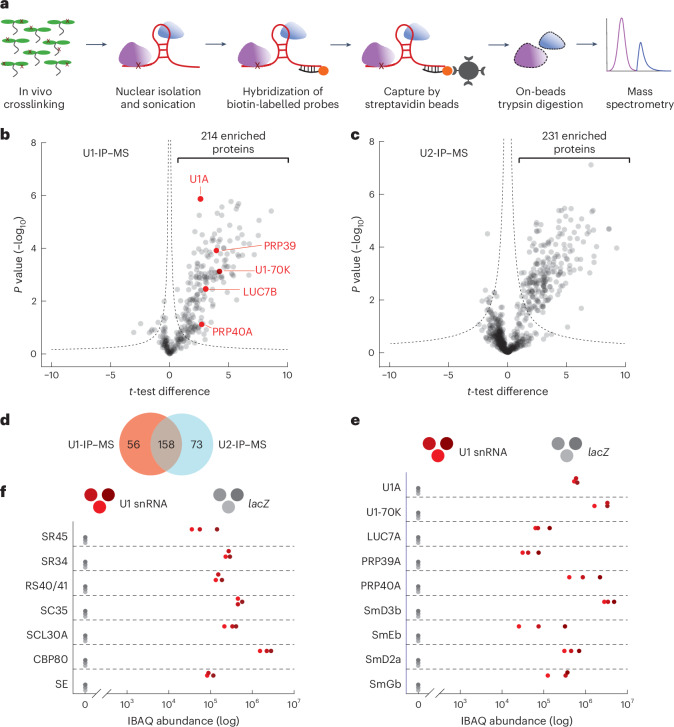
Identification of *Arabidopsis* U1 snRNP-associated proteins by U1-IP–MS. **a**, Schematic representation of the U1-IP–MS experiment. **b**,**c**, Analysis of U1 snRNA-associated (**b**) and U2 snRNA-associated (**c**) proteins identified by IP–MS. Volcano plot of three biological replicates showing significantly enriched proteins immunoprecipitated with a U1 (**b**) or U2 (**c**) antisense oligonucleotide compared with a *lacZ* oligonucleotide. For this, a two-sided *t*-test was performed between U1-IP–MS and *lacZ*-IP–MS (**b**) and between U2-IP–MS and *lacZ*-IP–MS (**c**). The hyperbolic curve indicates the significant threshold with an FDR of 0.04 for U1-IP–MS and 0.05 for U2-IP–MS. Known U1-specific proteins are highlighted in red (**b**). **d**, Venn diagram depicting the overlap between significantly enriched proteins in U1-IP–MS and U2-IP–MS experiments. **e**,**f**, Abundance of specific proteins in U1-IP–MS experiments. The three red and grey dots represent iBAQ values of three biological replicates using the U1 or the *lacZ* antisense oligonucleotide, respectively. Proteins known to be part of the U1 snRNP (**e**) and selected proteins that function in splicing and RNA processing (**f**) are shown.

Despite the importance of the U1 snRNP in splicing and beyond, very little is known about the composition of the U1 snRNP or associated proteins and complexes in plants. To identify the proteins associated with a plant U1 snRNP complex, we applied ‘comprehensive identification of RNA-binding proteins by mass spectrometry’, which has been successfully applied to isolate proteins associated with the U1 snRNA or other non-coding RNAs^37^. We used a biotinylated U1 snRNA antisense probe to purify the *Arabidopsis* U1 snRNP and analysed the purified sample by mass spectrometry (U1-IP–MS; Fig. 1a). A short-distance crosslinking agent, formaldehyde, was used to preserve transient interactions of the U1 snRNP with other proteins and complexes during the purification procedure. To test whether we can indeed observe dynamic interactions with this approach as well, we performed a similar experiment with an antisense oligonucleotide specific for the U2 snRNA (U2-IP–MS). The U1 snRNP, as part of the commitment complex, recruits the U2 snRNP for the formation of the A complex. Hence, we would expect a partially overlapping set of proteins associated with the U1 and the U2 snRNAs. As a negative control, we performed an immunoprecipitation followed by mass spectrometry (IP–MS) experiment using an antisense *lacZ* oligonucleotide, the sequence of which is not expected to bind any RNA encoded in the *Arabidopsis* genome. Three biological replicates were prepared for each IP–MS experiment. In total, we were able to identify 908 proteins by MS (Fig. 1b, complete lists in Supplementary Data 1).

**Extended Data Fig. 1:**
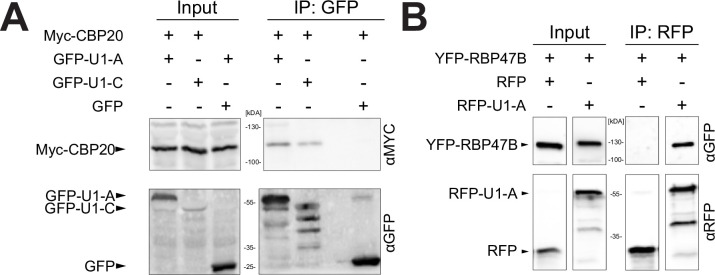
Interaction test between U1 snRNP components and CBP20 and RBP47B. **a**: U1-A or U1-C translationally fused to GFP was co-expressed with MYC-tagged CBP20 in *Nicotiana benthamiana* plants for transient protein expression. GFP alone served as a negative control. Proteins were isolated and immunoprecipitated using a GFP-affinity matrix. Input and immunoprecipitated fractions (IP) were subjected to protein blot analysis using GFP- or –MYC specific antibodies. Each experiment was repeated three times independently with similar results. **b**: U1-A translationally fused to RFP was co-expressed with YFP-tagged RBP47 in *Nicotiana benthamiana* plants for transient protein expression. RFP alone served as a negative control. Proteins were isolated and immunoprecipitated using an RFP-affinity matrix. Input and immunoprecipitated fractions (IP) were subjected to protein blot analysis using RFP- or -YFP specific antibodies. Each experiment was repeated two times independently with similar results. Source data

**Extended Data Fig. 2:**
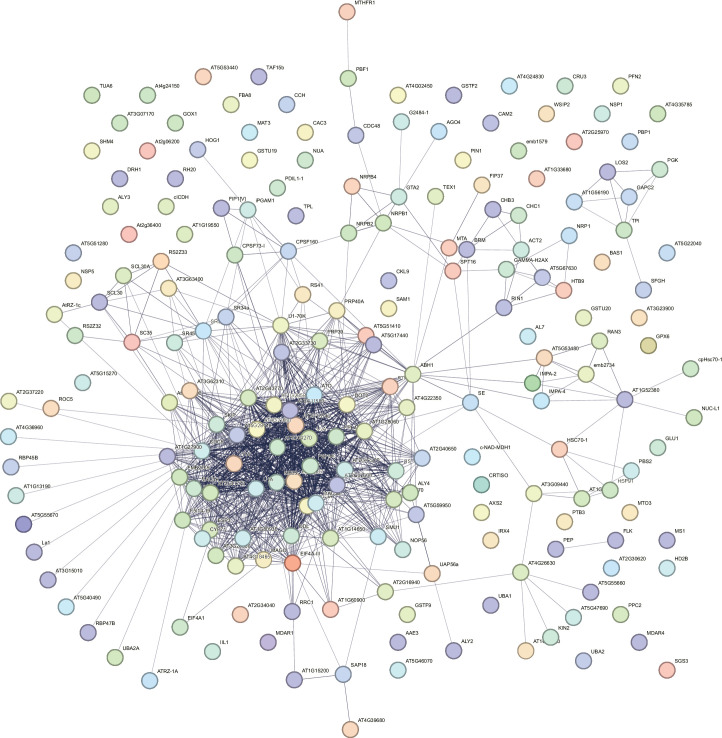
String analysis reveals known interactions between significantly enriched proteins in the U1-IP-MS experiment. We applied the following parameter for the String analysis: interaction sources: Textmining, Experiments, Databases, minimum required interaction score: high confidence.

We found 214 proteins significantly enriched in IPs with the U1 snRNA antisense probe (Fig. 1b and Supplementary Table 1). With the U2 snRNA antisense probe, we retrieved 231 significantly enriched proteins (Fig. 1c and Supplementary Table 2). Some 158 proteins were found to be associated with both the U1 and U2 snRNA antisense probe, while 56 and 73 proteins were specifically associated with the U1 and U2 snRNA antisense probe, respectively (Fig. 1d and Supplementary Table 3). The large number of proteins that co-purified with the U1 and U2 snRNA antisense probes indicates that our approach was able to capture transient interactions that occurred, for example, during the formation of the A complex. The effectiveness of the U1 snRNA IP is further supported by the successful enrichment of known U1 snRNP core and accessory components; we found known U1 snRNP components such as U1-A, U1-70K, LUC7B, PRP39, PRP40A and Sm core proteins (SmB, SmD1, SmE, SmG) (Fig. 1b,e and Supplementary Table 1). Not a single peptide of the above-mentioned proteins was retrieved in the control IP experiments using the *lacZ* antisense probe (Fig. 1e). U1-IP–MS also enriched splicing factors, many of which are known to interact with the U1 snRNP including the serine/arginine-rich (SR) proteins SR45, SR34, RS40/41, SC35 and SCL30A, as well as components of the MOS4-associated complex (MAC), which is the homologue of the metazoan Nineteen-complex^38–40^ (Fig. 1f and Supplementary Table 1). We also retrieved other splicing-related proteins, such as SERRATE and the nuclear cap-binding complex (nCBC), consisting of the two subunits, CBP80 and CBP20, or the stress granule RRM-domain-containing protein RPB47B^40–42^. We additionally confirmed the interaction between CBP20 with the U1 SNP core proteins, U1A and U1C, by co-immunoprecipitation (Extended Data Fig. 1a). In addition, RBP47B co-immunoprecipitated with U1-A, suggesting that the interactions identified through U1-IP–MS are indeed authentic (Extended Data Fig. 1b). A STRING analysis for functional and physical interactions among proteins revealed a tight interaction network among the U1 snRNA-associated proteins (*P* < 1.0 × 10^−16^; Extended Data Fig. 2)^43^. Enrichment analysis showed that U1 snRNA-associated proteins often feature RNA binding motifs, helicases and WD40 repeats (Supplementary Data 2). Although U1 snRNA-associated proteins were mainly involved in splicing, gene ontology analysis revealed that also other biological processes such as microRNA (miRNA) processing, RNA transport, RNA silencing or the regulation of transcription were significantly enriched among U1 snRNA-associated proteins (Supplementary Table 4 and Data 2). Taken together, the U1-IP–MS experiment revealed more than 200 proteins statically or dynamically associated with the U1 snRNA, and our results suggest functions of the plant U1 snRNP beyond splicing.

### The U1 snRNP associates with cleavage and polyadenylation factors

**Fig. 2:**
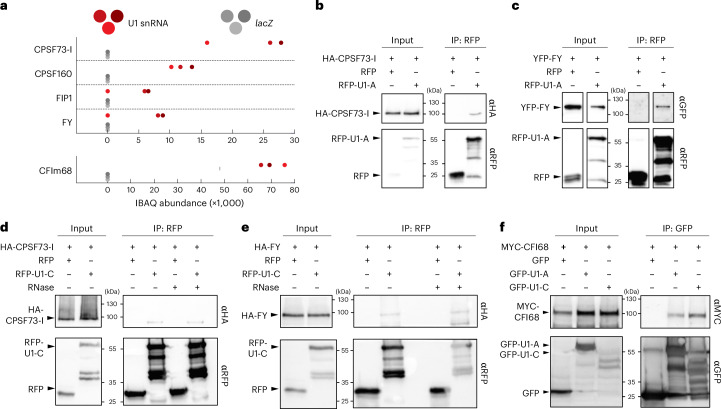
The U1 snRNP core components, U1-A and U1-C, associate with mRNA cleavage and polyadenylation factors. **a**, Abundance of CPAFs in U1-IP–MS experiments. The three red and grey dots represent iBAQ values of three biological replicates using the U1 or the *lacZ* antisense oligonucleotide, respectively. **b**,**c**, U1-A translationally fused to RFP was co-expressed with HA-tagged CFSF73-I (**b**) or YFP-tagged FY (**c**) in *N. benthamiana* plants for transient protein expression. RFP alone served as a negative control. Proteins were isolated and immunoprecipitated using an RFP-affinity matrix. Input and immunoprecipitated fractions (IP) were subjected to protein blot analysis using RFP-, HA- and G/YFP-specific antibodies. Each experiment was repeated two times independently with similar results. **d**,**e**, U1-C translationally fused to RFP was co-expressed with HA-tagged CFSF73-I (**d**) or FY (**e**) in *N. benthamiana* plants for transient protein expression. RFP alone served as a negative control. Proteins were isolated and immunoprecipitated using an RFP-affinity matrix in the presence or absence of RNase A. Input and immunoprecipitated fractions (IP) were subjected to protein blot analysis using RFP- and HA-specific antibodies. Each experiment was repeated three times independently with similar results. **f**, MYC-CFI68 was transiently co-expressed with GFP-U1-A, GFP-U1-C or GFP in *N. benthamiana* plants. After immunoprecipitation using a GFP-affinity matrix, the isolated proteins were subjected to protein blot analysis. GFP- and MYC-specific antibodies were used for the detection of the tagged proteins. Each experiment was repeated three times independently with similar results. Source data

Crosstalk between mRNA CPAFs and the spliceosome, including the U1 snRNP, has been well documented in metazoans^27,28,36,44–48^; however, this association and its functional relevance remain largely unexplored and unidentified within plant species. Among the 214 U1 snRNA-associated proteins identified by U1-IP–MS, we found several CPAFs, including components of the cleavage and polyadenylation specificity factor (CPSF) and the cleavage factor I (CFI) complex (Fig. 2a and Supplementary Table 1). The CPSF recognizes the polyadenylation signal (PAS) (in metazoans, AAUAAA), cleaves the pre-mRNA and recruits poly(A)polymerases for polyadenylation^49^. CPSF acts in concert with other complexes: cleavage stimulation factor (CstF), CFI and cleavage factor II^50^. These complexes bind additional *cis*-regulatory elements, upstream sequence elements (USE) and downstream sequence elements (DSE). While the canonical *cis* motifs involved in CPA are less well-conserved in plants compared with metazoans, the proteins involved in CPA are highly conserved^51–53^. The CPSF consists of several subunits: CPSF73, CPSF160, CPSF30, WDR33, FIP1 and CPSF100. CPSF73 functions as an endonuclease and is encoded by two essential genes in *Arabidopsis*, CPSF73-I and CPSF73-II^54–56^. FY is the WDR33 homologue in *Arabidopsis* and recognizes the PAS in concert with CPSF160 (refs. ^57–59^). CFI consists of four units of different combinations of CFI25, CFI59 and CFI68 (refs. ^60,61^). Mutations in the CPSF or CFI components show mild to drastic phenotypic alterations and changes in mRNA CPA^54–56,60,62–67^.

We found CPSF73-I, CPSF160 and FIP1 among the 214 significant proteins identified by U1-IP–MS, suggesting that U1 snRNP forms a high-order complex with the CPSF (Fig. 2a). To check this notion, we tested whether protein components of the U1 snRNP co-immunoprecipitate with the CPSF. For this, we transiently co-expressed RFP-U1-A or RFP-U1-C together with HA-CPSF73-I fusion proteins and performed affinity purification using an anti-RFP-affinity matrix. HA-CPSF73-I co-purified with RFP-U1-A and RFP-U1-C, but not RFP, which suggests a physical interaction between proteins of the U1 snRNP and the CPSF (Fig. 2b,d). The U1-IP–MS also contained peptides for two other CPSF subunits, FY and CPSF30, but failed to reach the significance threshold (Fig. 2a and Supplementary Data 1). Still, we also found that YFP-FY co-immunoprecipitated with RFP-U1-A and RFP-U1-C, but not with RFP (Fig. 2c,e). These co-immunoprecipitations of HA-CPSF73-I with RFP-U1-A and RFP-U1-C and YPF-FY with RFP-U1-A and RFP-U1-C, as well as the presence of CPSF73-I, CPSF160 and FIP1 in the U1-IP–MS experiments, strongly support the physical interaction between the *Arabidopsis* U1 snRNP and CPSF. To test whether the interaction between the U1 snRNP and the CPSF depends on RNA, we analysed the interaction between RFP-U1-C and HA-CPSF73-I or HA-FY in the presence of RNase A. CPSF73-I and FY co-immunoprecipitated in U1-C pull down, suggesting that the interaction between the U1 snRNP and the CPSF is not RNA-dependent (Fig. 2d,e). The U1-IP–MS also retrieved a component of the CFI complex, CFIm68, which binds to the USE (Fig. 2a). Interaction between CFIm68 and the U1 snRNP core proteins U1-A and U1-C were also detected in co-immunoprecipitation experiments (Fig. 2f). This suggests that the U1 snRNP may interact with other components involved in CPA, in addition to CPSF components.

### U1 snRNP is crucial for plant growth and transcriptome integrity

**Fig. 3:**
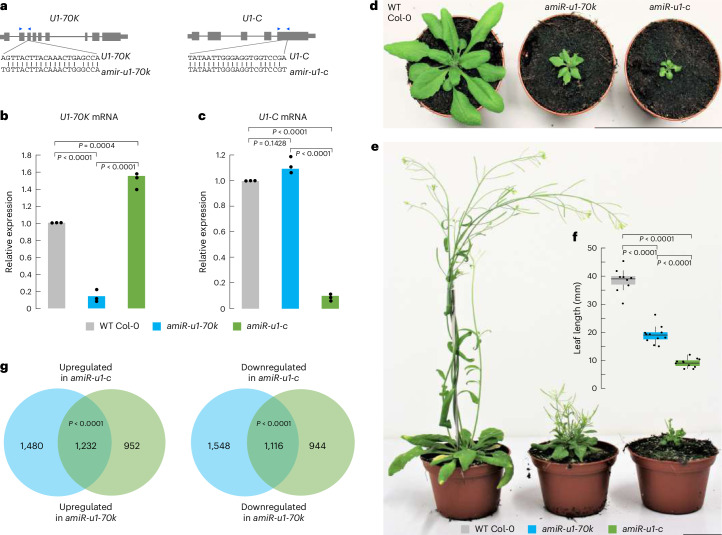
Knockdown of two U1 snRNP core components, U1-70K and U1-C, drastically affects plant development and gene expression. **a**, Gene models of *U1-70K* and *U1-C* and regions used for the design of amiRNAs. The blue arrowheads indicate the position of PCR primers used for RT–qPCR in Fig. 2b,c. **b**,**c**, RT–qPCR analysis of *U1-70K* (**b**) and *U1-C* (**c**) levels in 7-day-old WT, *amiR-u1-70k* and *amiR-u1-c* seedlings. The bars indicate the average relative expression in three biological replicates and the dots represent the three independent measurements. Statistical significance was tested using one-sided analysis of variance followed by Tukey’s honestly significant difference test. **d**,**e**, Phenotypes of WT, *amiR-u1-70k* and *amiR-u1-c* plants grown for 21 days (**d**) or 56 days (**e**) under long-day (16 h light/8 h darkness) conditions. **f**, Leaf length of WT, *amiR-u1-70k* and *amiR-u1-c* plants, measured after 21 days. In this boxplot, the dots represent the individual leaf length measurements (at least ten plants for each genotype) and the black lines inside the boxes represent the median length. The upper and lower boundaries are indicated by the coloured boxes showing the 25th and 75th quartiles, and the black whiskers represent the 5th and 95th percentiles. Statistical significance was tested using one-sided analysis of variance followed by Tukey’s honestly significant difference test for pairwise comparison. **g**, Venn diagrams depicting the overlap of differentially expressed genes in *amiR-u1-70k* and *amiR-u1-c* compared with WT. Expression was determined by RNA-seq and differentially expressed genes were considered as all genes that significantly differed between the WT and U1 knockdown line (*P*_adj_ < 0.05). Significance was tested using one-sided hypergeometric overlap test.

To study the functions of the *Arabidopsis* U1 snRNP and its possible function beyond splicing, such as mRNA CPA, the research community lacks plants with reduced levels of core U1 proteins, which cause drastic phenotypic alterations. To address this issue, we generated U1 snRNP knockdown lines using artificial microRNAs (amiRNAs) that target the mRNAs of the two U1 core subunits, U1-70K and U1-C (referred to as *amiR-u1-70k* and *amiR-u1-c*; Fig. 3a), respectively. This resulted in a reduction of their mRNA levels to ~10% of that found in wild-type (WT) plants (Fig. 3b,c). We speculated that targeting two different genes encoding proteins forming a common complex would result in similar mutant phenotypes. Indeed, the knockdown of the core U1 subunits U1-C and U1-70K resulted in plants exhibiting pleiotropic defects in plant development, including dwarfism and abnormal leaf development (Fig. 3d–f). While these plants produced a reduced number of seeds, their ability to develop viable seeds despite their extreme phenotype makes them a valuable genetic tool for the entire research community. The altered phenotypes were observed for the vast majority of primary transformants, with the knockdown of *U1-C* always leading to slightly more severe phenotypic alterations (Fig. 3d–f).

**Extended Data Fig. 3:**
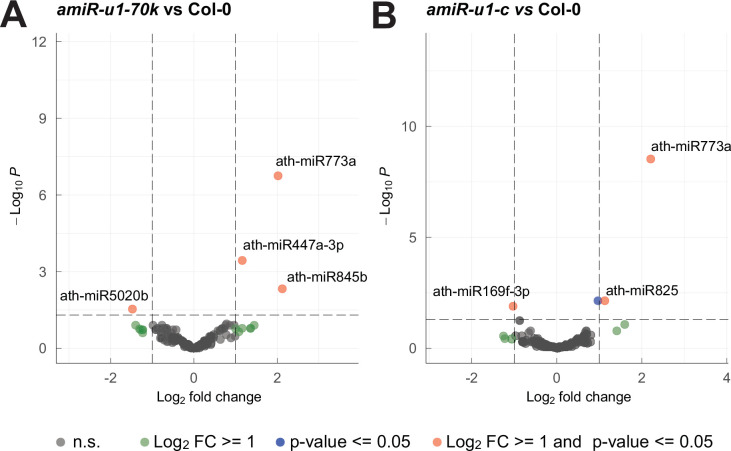
miRNA are not widely differentially expressed in U1 knockdown lines. **a, b**: miRNAs expression analysis by small RNA-seq from WT, *amiR-u1-70k*, and *amiR-u1-c* plants. Volcano plots show differentially expressed miRNAs in *amiR-u1-70k* (**a**) or *amiR-u1-c* (**b**) compared to WT Col-0 plants using three biological replicates. A total of 178 miRNAs were analyzed. Grey dots depict miRNAs not significantly changed (n.s.), green dots depict miRNAs with a log2 fold change ≥ 1, blue dots depict miRNAs with an FDR ≤ 0.05, and red dots depict miRNAs with a log2 fold change ≥ 1 and an FDR ≤ 0.05.

To determine whether the reduction of *U1-70K* and *U1-C* expression also had comparable effects on the transcriptome, we performed a short-read RNA-seq experiment using WT, *amiR-u1-70k* and *amiR-u1-c* plants with two to three replicate measurements. In total, we found 2,712 and 2,184 significantly upregulated and 2,664 and 2,060 significantly downregulated genes in *amiR-u1-70k* and *amiR-u1-c* lines, respectively, when compared with WT plants (Supplementary Data 3). A significant number of upregulated (1,232) and downregulated (1,116) genes overlap between *amiR-u1-70k* and *amiR-u1-c* plants (Fig. 3g), which further supports the idea that knocking down two different genes encoding proteins of the U1 snRNP results in similar molecular phenotypes. Because several reports suggest an involvement of the U1 snRNP components in miRNA biogenesis^14,68^, we also performed a small RNA-sequencing analysis with WT, *amiR-u1-70k* and *amiR-u1-c* plants. We did not observe any drastic change in miRNA accumulation in U1 knockdowns compared with WT (Extended Data Fig. 3). Although U1 accessory factors such as PRP40 fulfil important functions in miRNA biogenesis, the core U1 snRNP probably has only minor functions in general miRNA biogenesis.

**Fig. 4:**
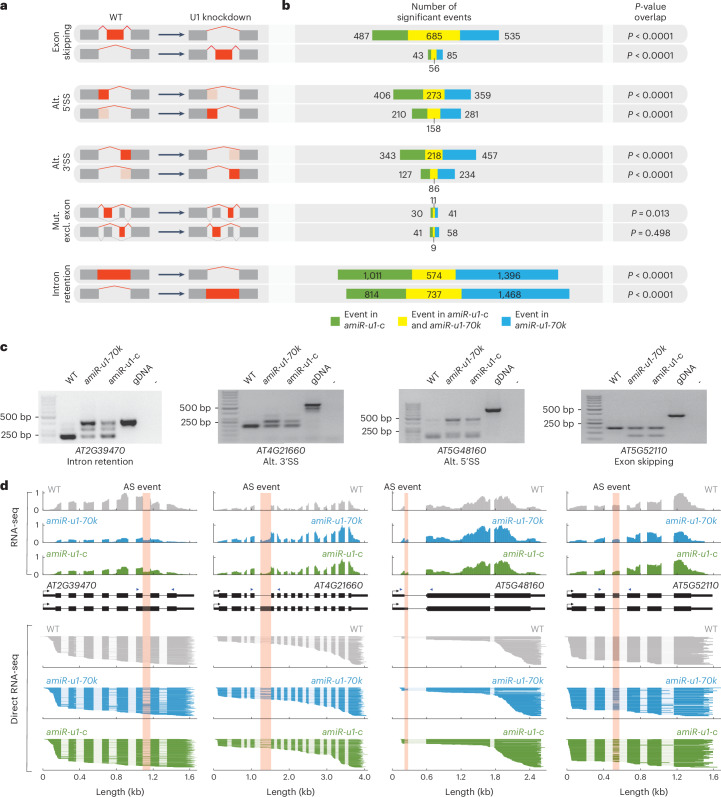
Knockdown of U1-*70K* or *U1-C* causes overlapping splicing defects. **a**,**b**, Changes in the splicing pattern were calculated on the basis of RNA-seq data from WT (**a**) and *amiR-u1-70k* and *amiR-u1-c* (**b**) plants using rMATS. Splicing changes were subcategorized into exon skipping, alternative (alt.) 5′SS, alternative 3′SS, mutually exclusive exons (mut. excl. exon) and intron retention. A schematic representation of the different splicing changes is shown in **a**. The numbers of significantly differential alternative splicing events are shown. Significance of overlaps in splicing changes in *amiR-u1-70k* and *amiR-u1-c* plants was tested using one-sided hypergeometric overlap test. **c**, RT–PCR analysis of selected alternative splicing events detected in the RNA-seq dataset. Primers used for amplification were designed to flank the splicing event. Position of primers is depicted by blue arrowheads in **d**. **d**, ONT direct RNA-seq reads aligned to the genes that produced alternative spliced RNAs (**c**). The coverage plot of one representative replicate of the RNA-seq dataset used for rMATS analysis (**a**,**b**) is shown. Pink boxes indicate the alternative splicing (AS) events detected by rMATS.

Because U1-70K and U1-C probably fulfil key functions during splicing, we globally evaluated splicing changes in *amiR-u1-70k* and *amiR-u1-c* lines using the above-described short-read RNA-seq dataset and the rMATS software^69^. Alternative splicing events were grouped into different categories: exon skipping, alternative 5′SS or 3′SS, intron retention and mutually exclusive exons (Fig. 4a). U1 knockdown resulted in a large number of splicing defects, especially in intron retention (3,136 and 4,175 events in *amiR-U1-C* and *amiR-U1-70K*, respectively) and exon skipping (1,271 and 1,361 events in *amiR-U1-C* and *amiR-U1-70K*, respectively) (Fig. 4b and Supplementary Data 4). Especially interesting is the trend in *amiR-U1-70K* and *amiR-U1-C* lines to accumulate mRNAs that lack exons through exon skipping (Fig. 4b). U1 knockdowns in metazoans or *Arabidopsis* mutants in U1 accessory factors such as *LUC7* show very similar patterns in splicing defects^19,24,70^, which is probably due to the altered connection between the U1 and U2 snRNP. Percentages of 58.3 and 54.0% of the exon skipping events and 41.9 and 31.5% of the intron retention events were shared between the *amiR-u1-70k* and *amiR-u1-c* lines, which again strongly suggests that both independent knockdown lines have highly similar defects (Fig. 4b and Supplementary Data 4). We also observed 5′SS and 3′SS splicing changes, which were significantly overlapping between *amiR-u1-70k* and *amiR-u1-c* lines, suggesting that an intact U1 snRNP is essential for splicing fidelity in general (Fig. 4b and Supplementary Data 4). The changes in alternative splicing were not due to changes in mRNA expression, because we found ~80% of all splicing defects in genes that were not differentially expressed in *amiR-u1-70k* and *amiR-u1-c* lines compared with WT (Extended Data Fig. 5 and Supplementary Data 5). To exemplarily validate splicing defects that were detected using rMATS, we performed PCR with reverse transcription (RT–PCR) with different biological replicates and primers flanking regions of alternative splicing events, which were found in both U1 knockdown lines (Fig. 4c). In addition, we performed Oxford Nanopore Technologies (ONT) direct RNA-seq with additional biological replicates of WT, *amiR-u1-70k* and *amiR-u1-c* plants. While the total number of reads obtained by direct RNA-seq was too low to perform global splicing analysis, the coverage plots of selected splicing events clearly confirmed the short-read RNA-seq analysis (Fig. 4d).

**Extended Data Fig. 4:**
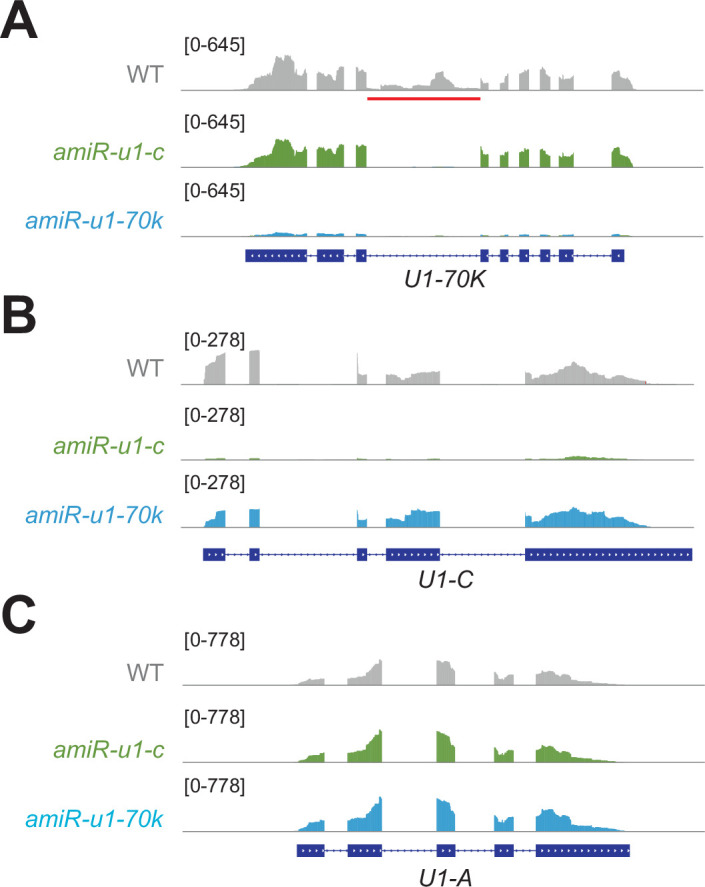
*U1-C* expression affect the splicing of *U1-70K.* **a-c**: The figure depicts the gene models of *U1-70K* (**a**), *U1-C* (**b**) and *U1-A* (**c**) and the corresponding RNA-seq coverage plots in WT, *amiR-u1-70k*, and *amiR-u1-c*. The red line indicates an intron in *U1-70K* known to be subjected to alternative splicing.

**Extended Data Fig. 5:**
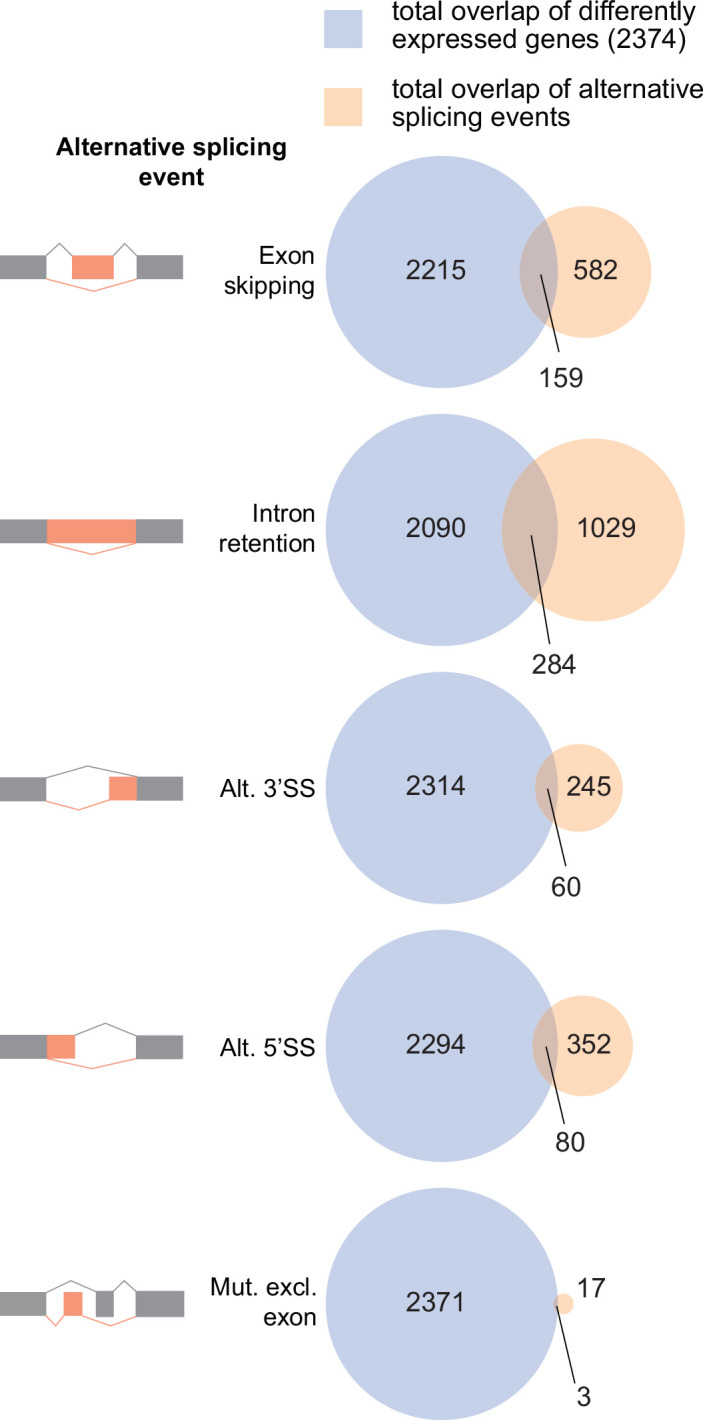
Overlap between differentially expressed genes and differentially spliced genes in *amiR-u1-70k*, and *amiR-u1-c.* Venn digramms depict the overlap between differentially expressed genes in *amiR-u1-70k*, and *amiR-u1-c* lines and differentially spliced genes found in *amiR-u1-70k*, and *amiR-u1-c*. Splicing changes were subcategorized into exon skipping, alternative 5′splice site (alt. 5′SS), alternative 3′splice site (alt. 3′SS), mutually exclusive exons (mut. excl. exon), and intron retention.

A potentially interesting finding is that *U1-C* might regulate the expression of *U1-70K* via alternative splicing. Plant *U1-70K* genes produce an additional, non-functional mRNA isoform that retains the sixth intron and exhibits features such as long, intron-containing 3′-UTRs, which probably subjects this isoform to the non-sense mediated mRNA decay (NMD) pathway^71,72^. In humans, U1-C regulates the production of non-functional U1-70K subjected to degration via the NMD pathway. This might hint at an evolutionarily conserved intra-U1-snRNP-specific regulatory feedback loop to balance the production of functional U1 snRNPs^73^. We could easily detect the *U1-70K* isoform with the retained sixth intron in WT plants but found a strong reduction of this isoform in *amiR-u1-c* plants (Extended Data Fig. 4). This suggests that U1-C, directly or indirectly, affects the splicing of *U1-70K* and production of functional *U1-70K* mRNA in *Arabidopsis* and might explain the somewhat higher levels of *U1-70K* mRNA in the *amiR-u1-c* line (Fig. 3b).

Taken together, these results show the importance of the U1 snRNP in maintaining the normal development of plants and highlight the significance of the U1 snRNP for transcriptome integrity and splicing fidelity. Furthermore, U1 knockdown lines might serve as a powerful tool for studying functions of the *Arabidopsis* U1 snRNP beyond splicing.

### The *Arabidopsis* U1 snRNP features telescripting function

**Fig. 5:**
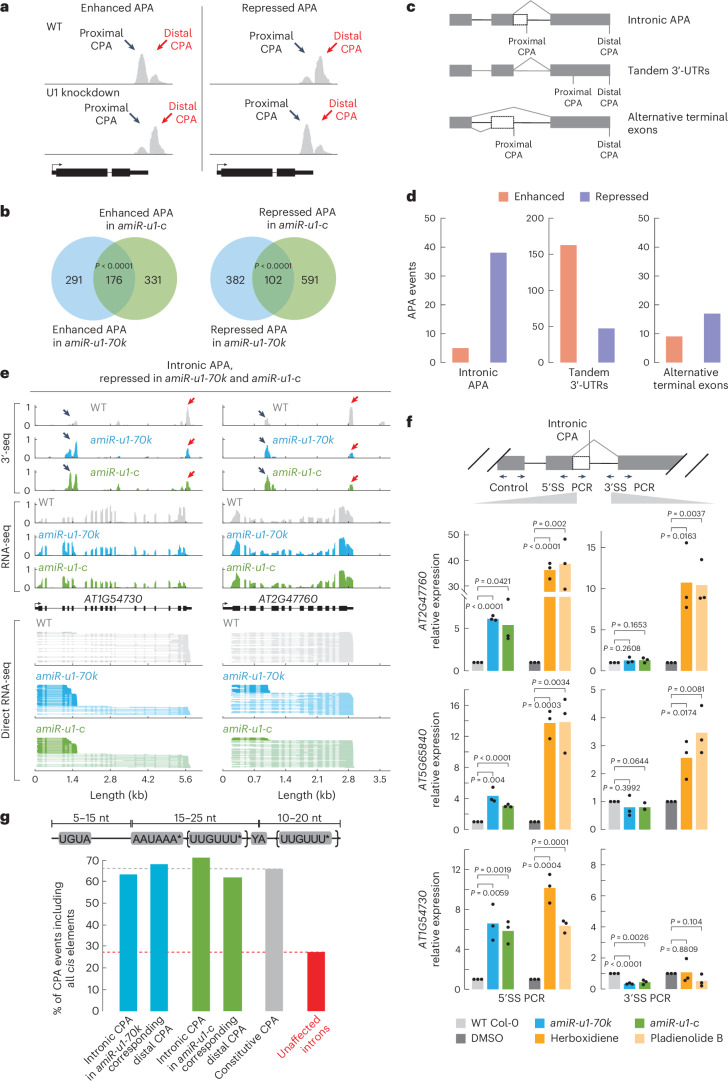
The U1 snRNP regulates alternative polyadenylation in *Arabidopsis*. **a**, A schematic representation of enhanced and repressed APA events. In enhanced APA events, the proximal CPA site is preferentially utilized. In repressed APA events, the distal CPA site is preferentially utilized. Black arrows indicate the proximal CPA and red arrows indicate the distal CPA. **b**, Polyadenylation sites were detected by 3′-end sequencing of RNAs (3′-seq) experiments using RNA isolated from 7-day-old WT, *amiR-u1-70k* and *amiR-u1-c* seedlings. Venn diagrams depict the overlap of enhanced or repressed APA events in *amiR-u1-70k* and *amiR-u1-c* when compared with WT. Significance was tested using a one-sided hypergeometric overlap test. **c**, A schematic representation of three different types of APA: intronic APA, tandem 3′-UTRs and alternative terminal exons. **d**, Number of different APA events detected in both *amiR-u1-70k* and *amiR-u1-c* plants, when compared with WT. Intronic APA, tandem 3′-UTR and alternative terminal exons were further divided into enhanced and repressed events. **e**, Two examples of intronic APA events that are repressed in *amiR-u1-70k* and *amiR-u1-c* plants. The figure depicts the gene models and the corresponding coverage plots for 3′-seq, RNA-seq and direct RNA-seq. **f**, RT–qPCR analysis of 7-day-old WT, *amiR-u1-70k* and *amiR-u1-c* seedlings, or WT seedling grown in liquid culture for 7 days and treated with DMSO (mock), berboxidiene or pladienolide B. PCR was performed with oligonucleotides spanning the 5′SS or 3′SS and results were normalized to an internal control. The bars indicate the average relative expression in three biological replicates and the dots represent the three independent measurements. A one-sided *t*-test was applied. **g**, Analysis of the co-occurrence of three specific *cis*-elements in different genomic features. Existence of UGUA, AAUAAA (allowing one mismatch except AAAAAA) and a UUGUUU motif (allowing one mismatch except UUUUUU) before or after the cleavage sites were analysed. Genomic features were chosen as follows: introns that are pCPAed in *amiR-u1-70k* or *amiR-u1-c* (and the corresponding distal CPA site), CPA sites in genes exhibiting only a single CPA site (constitutive CPA) and all introns that are not pCPAed (unaffected introns).

Given the association of the *Arabidopsis* U1 snRNP with CPSF components, we investigated its potential role in regulating CPA. To address this, we utilized the above-described U1 knockdown lines and performed 3′-end mRNA sequencing with WT, *amiR-u1-70k* and *amiR-u1-c* plants. In this dataset, we could detect ~18,000 genes that undergo alternative cleavage and polyadenylation (APA), with the majority of genes exhibiting more than four CPA sites. Changes in the usage of the CPA site were categorized into enhanced and repressed APA events and for simplification, only the two most abundant CPA sites were considered. The term ‘enhanced APA’ refers to cases where proximal CPA site usage is higher in WT than in the U1 knockdown lines (Fig. 5a), while the term ‘repressed APA’ indicates that the usage of the proximal CPA site in WT is lower than in the U1 knockdown lines (Fig. 5a). We found 467 enhanced and 484 repressed APA events in *amiR-u1-70k* plants, and 507 enhanced and 693 repressed APA events in *amiR-u1-c* plants (Fig. 5b and Supplementary Data 6). Among these, a significant number of enhanced (176, *P* = 6.71 × 10^−67^) and repressed (102, *P* = 1.24 × 10^−6^) APA events were shared between *amiR-u1-c* and *amiR-u1-70k* lines, suggesting that U1-C and U1-70K serve similar functions in mRNA cleavage and polyadenylation (Fig. 5b). We cannot entirely exclude the possibility that changes in CPA site usage are indirectly caused by expression changes or alternative splicing of genes encoding *CPAFs* in U1 knockdowns. We found five known genes involved in CPA that are differentially regulated in U1 knockdown lines (Supplementary Data 7); however, given the physical association of the U1 snRNP, CPSF and CFI components, we favour the idea that the U1 is directly affecting CPA through protein–protein interactions.

**Extended Data Fig. 6:**
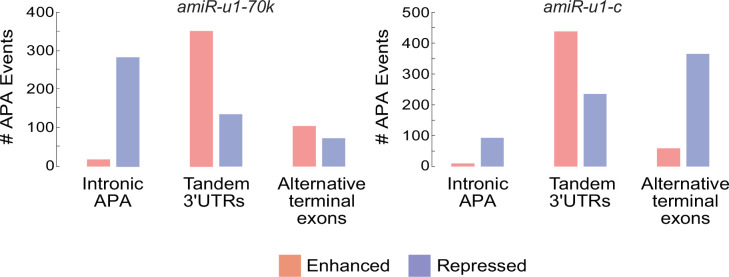
APA events in *amiR-u1-70k* and *amiR-u1-c* plants*.* Number of different APA events detected in *amiR-u1-70k* and *amiR-u1-c* plants, when compared to WT. Tandem 3′UTRs, intronic APA, and alternative terminal exons were further divided into enhanced and repressed events. The overlap of the different APA events is depicted in Fig. 5e.

We further categorized the APA events into three different categories (Fig. 5c)^74^: First, when proximal and distal CPA sites are located in the same terminal exon, it is designated as a ‘tandem 3′-UTR’ APA event. Second, ‘intronic APA’ events refer to cases in which the proximal CPA event resides in introns. Thus, this category includes premature cleavage and polyadenylation (pCPA) events generated by the lack of telescripting. Third, ‘alternative terminal exon’ events refer to APA events in which the proximal CPA site is located in a skipped exon. We observed interesting trends in ‘intronic APA’ events and ‘tandem 3′-UTR’ APA events; however, no pronounced trend was found in the alternative terminal exon category for *amiR-u1-70k* and *amiR-u1-c* plants (Fig. 5d, Extended Data Fig. 6 and Supplementary Data 6).

Both U1 knockdown lines exhibited more repressed intronic APA events, indicating that the intronic proximal CPA sites were utilized more frequently than the distal CPA sites in U1 knockdown lines (Extended Data Fig. 6 and Supplementary Data 6). Intronic APA events significantly overlapped (38 events, *P* = 1.19 × 10^−24^) between *amiR-u1-70k* (279 events) *and amiR-u1-c* lines (93 events), suggesting that both U1 components target a common set of genes for this type of APA regulation (Fig. 5d and Supplementary Data 6). In addition, we detected an accumulation of shorter-transcript isoforms for the selected significantly repressed intronic APA events by ONT direct RNA-seq (exemplified in Fig. 5e). While these shorter transcripts were also detectable in WT plants, they accumulated to higher levels in both U1 knockdown lines (Fig. 5e). To further validate these results, we performed quantitative PCR with reverse transciption (RT–qPCR) experiments with primers specifically amplifying a fragment that spans the 5′SS and a fragment spanning the 3′SS. In addition, control primers were used to amplify all mRNA isoforms generated from the gene. For all genes tested, mRNA containing the 5′SS accumulated in U1 knockdowns compared with WT, while mRNAs containing the 3′SS were unchanged or even less abundant (Fig. 5f). These results suggest that *Arabidopsis* genes can generate shorter mRNAs through pCPA in introns, but that the *Arabidopsis* U1 snRNP represses the usage of pCPA sites, akin to the telescripting function of the U1 snRNP in metazoans.

We aimed to determine whether pCPA is specifically due to loss of U1 snRNP function or if it is a secondary effect of reduced splicing efficiency. To test this, we inhibited the function of the SF3B subunit of the U2 snRNP by application of pladienolide B or herboxidiene, two potent inhibitors in plants, and performed RT–qPCR^75,76^. Chemical inhibition of the U2 snRNP led to an increase in mRNAs containing the 5′SS and 3′SS for the genes *AT2G47760* and *AT5G65840* (Fig. 5f). These results suggest that pCPA in the introns is not simply an effect of decreased splicing efficiency, but that the *Arabidopsis* U1 snRNP conducts a distinct function (telescripting) compared with the U2 snRNP. In the case of *AT1G54730*, pladienolide B or herboxidiene caused an increase in mRNAs containing the 5′SS, but not in mRNAs containing the 3′SS. These results suggest that for some introns, inhibiting splicing efficiency, regardless of reducing U1 or U2 snRNP, leads to pCPA.

**Extended Data Fig. 7:**
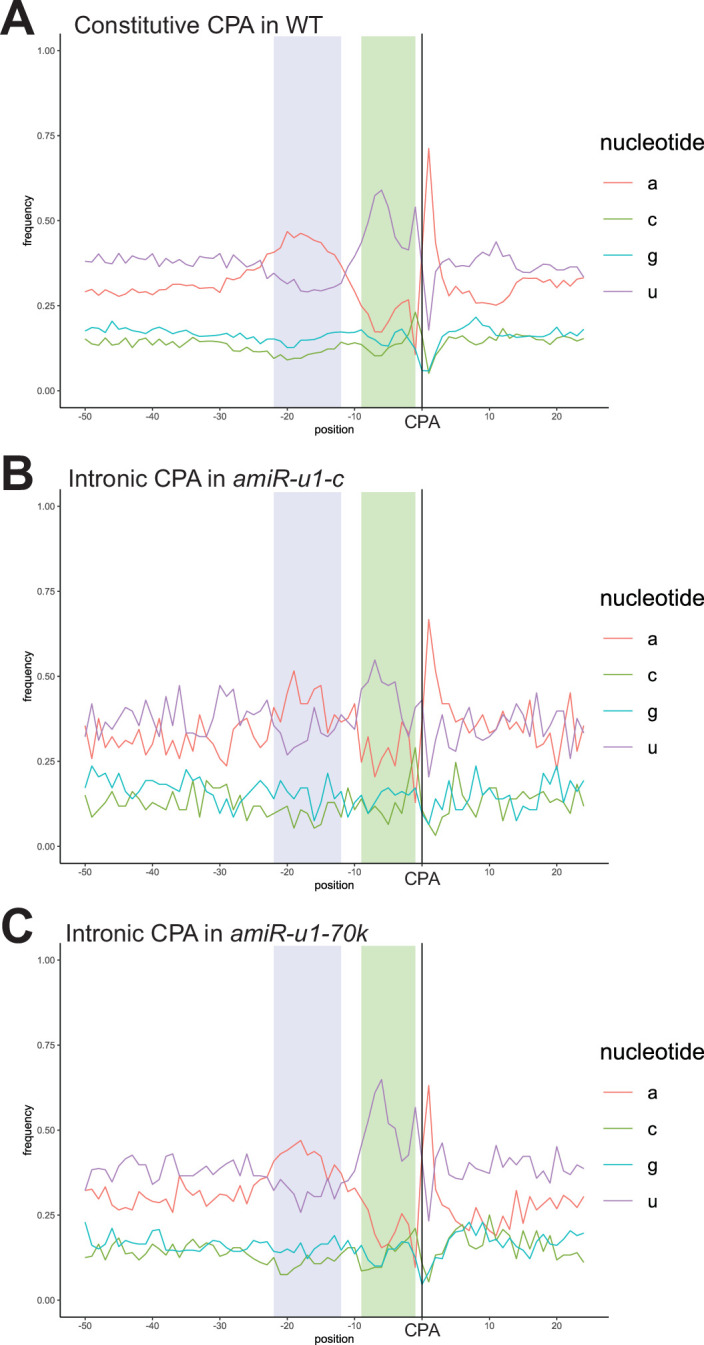
Nucleotide composition around CPA sites. **a-c:** The plots depict the nucleotide composition 50 nucleotide downstream and 25 nucleotides upstream of CPA sites for constitutive CPA sites in WT plants (**a**), intronic CPA sites in *amiR-u1-c* (**b**) and intronic CPA sites in *amiR-u1-70k* (**c**). An A-rich region (probably reflecting the PAS, highlighted in blue) and an U-rich region (probably reflecting the DSE highlighted in green) are marked.

To further analyse specific sequence features of introns that are subjected to pCPA in U1 knockdowns, we analysed nucleotide composition around cleavage sites. We observed a similar nucleotide distribution within pCPAed introns and constitutive CPA at the 3′-end of genes, namely, a U-rich region directly upstream and downstream of the cleavage site, and an A-rich region (Extended Data Fig. 7). To investigate specific motifs, we screened for the existence of canonical *cis*-elements important for polyadenylation: a downstream CFI binding site (UGUA), a downstream CPSF binding site (AAUAAA with one excepted alteration) and an upstream or downstream-located U/UG motif bound by cleavage stimulation factor. Approximately 65% of all constitutive CPA sites contain all three *cis*-elements (Fig. 5g). Introns that are prematurely cleaved and polyadenylated upon U1 knockdown contained all three motifs in 60–70% of all cases, while only 27% of unaffected introns showed all *cis*-elements (Fig. 5g). This disparity underscores the critical role of U1 snRNP in safeguarding against unintended polyadenylation, particularly in introns that feature all canonical *cis*-elements.

### Selection of CPA sites by the U1 snRNP in 3′-UTRs

**Fig. 6:**
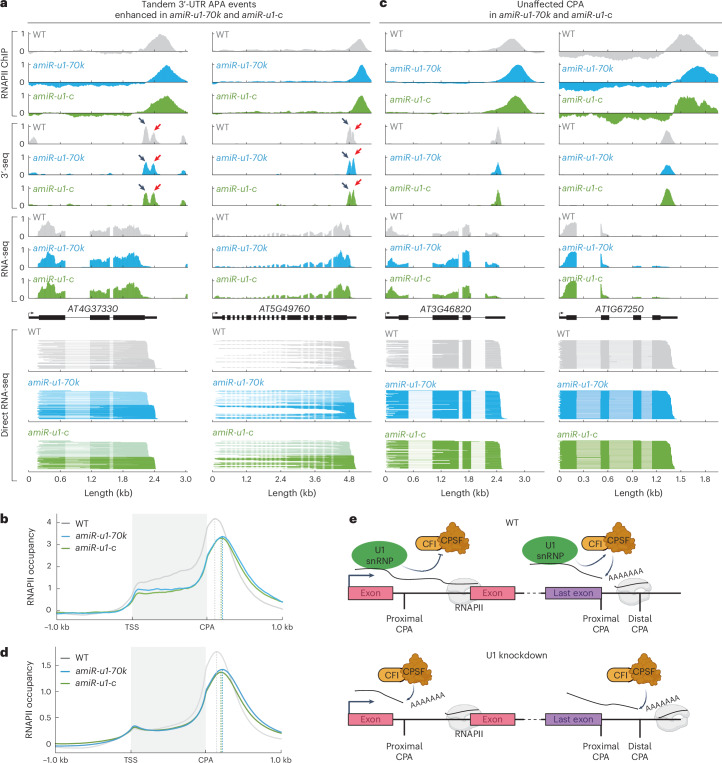
The U1 snRNP affects the distribution of RNA polymerase II at the 3′-end of genes. **a**, Two examples of a tandem 3′-UTR APA event enhanced in *amiR-u1-70k* and *amiR-u1-c*. The figure depicts the gene models and the corresponding coverage plots for polymerase II association (RNAPII ChIP), 3′-seq, RNA-seq and direct RNA-seq. **b**, Metaplot analysis of RNAPII binding to all genes exhibiting enhanced tandem CPA-site usage in WT, *amiR-u1-70k* and *amiR-u1-c* plants. **c**, Two examples of genes that exhibit a shift in RNAPII accumulation at the 3′-end, but the mRNAs of which are not subjected to APA. The figure depicts the gene models and the corresponding coverage plots for polymerase II association (RNAPII ChIP), 3′-seq, RNA-seq and direct RNA-seq. **d**, Metaplot analysis of RNAPII binding to all genes in WT, *amiR-u1-70k* and *amiR-u1-c* plants. **e**, Proposed model for the function of the U1 snRNP in RNA 3′ processing. The U1 snRNP associates with CPAFs. These interactions prevent premature intronic polyadenylation or ensure the usage of proximal polyadenylation sites in the last exons. In the absence of U1 snRNP function, intronic polyadenylation occurs and more distal polyadenylation sites are utilized in the last exons. Panel created with BioRender.com.

A closer look at the tandem 3′-UTR APA events revealed a different function of the U1 snRNP compared with its function in introns: both U1 knockdown lines exhibited more enhanced than repressed tandem 3′-UTR APA events, meaning that the WT prefers the proximal CPA site over the distal and the U1 mutants prefer the distal CPA site over the proximal. These events significantly overlapped between both knockdown lines (Fig. 5d and Supplementary Data 6). These results show that upon U1 knockdown in *Arabidopsis*, a subset of genes produces more of the longer isoform (exemplified in Fig. 6a).

Since we observed increased usage of distal CPA in terminal exons for some genes upon U1 knockdown, we asked whether RNAPII termination is also affected in U1 knockdown lines. Two models explain how transcription by RNAPII can be terminated. The allosteric model proposes that transcription of the PAS induces a structural change leading to termination. The torpedo model suggests that after RNA cleavage, the 5′-3′ exonuclease XRN2 rapidly degrades the remaining RNAPII-associated RNA, causing termination. More recent data suggest a combined model, in which structural changes facilitate catch-up of RNAPII by XRN2 (ref. ^77^). Consistently, the knockdown of factors such as human XRN2 or CPSF73 results in the production of longer transcripts and pile up of RNAPII further downstream of the CPA^77,78^. To test whether RNAPII association at terminal exons is affected by the U1 snRNP, we performed RNAPII chromatin immunoprecipitation (ChIP) experiments followed by sequencing (ChIP-seq) with WT and U1 knockdown lines. At genes with enhanced tandem 3′-UTR APA events in *amiR-u1-70k* and *amiR-u1-c*, RNAPII piled up downstream of the RNAPII peak at 3′-ends observed in WT (Fig. 6b, exemplified for individual genes in Fig. 6a). These results indeed suggest that RNAPII terminates more downstream at this subset of genes upon U1 knockdown. We observed a similar trend for many more genes, although the 3′-end sequencing did not detect any changes in CPA site usage between U1 knockdown lines and WT (exemplified in Fig. 6c). We therefore decided to investigate the RNAPII distribution among all *Arabidopsis* genes in WT and U1 knockdowns, regardless of whether more distal CPA sites in the terminal exon were utilized in U1 knockdowns. We observed a global shift of RNAPII to more distal sites and reduced accumulation of RNAPII at 3′-ends of genes in *amiR-u1-70k* and *amiR-u1-c* lines (Fig. 6d), which might suggest a more widespread role of the *Arabidopsis* U1 snRNP in CPA selection at 3′-ends. The reason we did not detect longer mRNAs when RNAPII terminates at more distal PAS in U1 knockdowns might be the lack of utilizable CPA sites or the fact that long 3′-UTRs of mRNAs trigger NMD^79,80^. Thus, the full consequences of U1 knockdown on the *Arabidopsis* transcriptome might only be detectable in U1 knockdown plants, which are also impaired in NMD or other RNA quality control mechanisms.

In summary, our results suggest at least two distinct functions of the U1 snRNP during CPA: First, the *Arabidopsis* U1 snRNP suppresses premature polyadenylation in gene bodies through telescripting. Second, the *Arabidopsis* U1 snRNP promotes the selection of proximal, canonical cleavage and polyadenylation sites at the 3′-end of mRNAs.

## Discussion

In this work, we report the identification of U1 snRNP-associated proteins in *Arabidopsis*. Using an RNA-centric approach, we enriched known U1 snRNP core and accessory components and identified proteins that may indirectly associate with the U1 snRNP, potentially hinting at their role in mRNA splicing or suggesting splicing-independent roles of the *Arabidopsis* U1 snRNP. In general, RNA-centric approaches for the isolation of RNA-containing protein complexes might be powerful tools for the detection of mRNPs. For the sake of fairness, one has to admit that the U1 snRNA is a very abundant RNA species, which alleviates RNA IP–MS experiments compared with less abundant RNA species. Although optimization might be required, RNA-centric approaches are an attractive tool to identify regulators of RNA processing, as they do not require generation of transgenics. For low-abundance RNAs, approaches involving RNA labelling might be better alternatives^81–85^.

Our *U1-70K* and *U1-C* knockdown lines exhibited much stronger phenotypic alterations compared with previously reported *U1-A* and *U1-70K* T-DNA insertion lines^20,21^. One possible explanation is that the analysed T-DNA mutants are not strong or true knockout alleles, especially for *U1-70K*, for which two different T-DNA lines with insertions at the 5′ and 3′-ends of the *U1-70K* gene were studied^20,21^. Another explanation for the lack of drastically altered phenotypes in U1 T-DNA lines might be functional redundancy. U1-A and U2B′, both of which bind to the U1 and U2 snRNA stem-loop, respectively, evolved from a single ancestral protein and exhibit functional redundancy in metazoans^86,87^. The sequences of *Arabidopsis* U1-A and U2B′ proteins are highly similar^88^, which might suggest some redundancy also in plants. Although U2B′ does not bind U1 snRNA under standard conditions^20,89^, U1 snRNA might be bound by U2B′ (or other sequence-related U1-A proteins) in *u1-a* mutants in vivo, thus explaining the lack of drastically altered phenotypes in *u1-a* mutants compared with our *U1-70K* and *U1-C* knockdown lines. Antisense morpholino oligonucleotides are a powerful tool to study U1 snRNP functions in human cell culture systems, but similar tools are currently unavailable in the plant research community^27^. Reduction of *U1-70K* and *U1-C* expression in *Arabidopsis* by amiRNAs resulted in overlapping phenotypic, RNA expression, splicing and CPA defects. Thus, these amiRNA lines (and further developments using tissue-specific or inducible promoters) become important tools for the future analysis of U1 functions in plants. While this study focuses on the overlapping functions of U1-70K and U1-C and thus most probably the entire U1 snRNP, some reports describe distinct functions outside their traditional roles in the U1 snRNP complex^90–93^. We also do not exclude the possibility that U1-70K and U1-C affect, for example, CPA in a U1 snRNP-independent manner. Nevertheless, our results, including overlapping CPA defects upon *U1-C* and *U1-70K* knockdown, the association of CPAFs with both U1-A and U1-C in co-IP experiments, and the association of CPAFs with the U1 snRNA detected by U1-IP–MS, along with data from metazoans, suggest that the U1 snRNP as a whole affects mRNA cleavage and polyadenylation. To study distinct functions of U1 core proteins, additional genetic tools need to be developed, such as the generation of hypomorphic U1 mutant alleles by genome editing.

The availability of U1-IP–MS data and U1 knockdown lines enabled us to study the function of the *Arabidopsis* U1 snRNP in mRNA 3′-end processing. Similar to the metazoan U1 snRNP, the *Arabidopsis* U1 snRNP interacts with RNA 3′-end processing complexes and possesses telescripting function to suppress intronic CPA sites. As in humans, no alteration in intronic CPA was found after chemical inhibition of the U2 snRNP for some genes, strongly supporting a distinct function of the U1 snRNP compared with the U2 snRNP^31^. Our results suggest that the presence of USE, PAS and DSE in introns plays an important role in intronic premature CPA and that these elements exist in 27% of all introns. Probably, not all of the genes are expressed at the developmental stage and tissue that we used for our investigation; therefore, many of these potential intronic CPA events might have escaped our analysis. Nevertheless, given the relatively high abundance of such *cis*-elements in introns, it is necessary to efficiently prevent premature CPA. For at least a fraction of these genes, the telescripting function of the U1 snRNP might fulfil this role. In humans, the 5′SS is important for telescripting within a 1 kb range, but additional cryptic 5′SS might be important for suppression of intronic CPAs in larger introns^28,31^. Because *Arabidopsis* introns are relatively short, cryptic 5′SS within introns might be of less importance; however, in plants with large introns, additional elements within introns might also play important roles in the production of full-length mRNAs^94,95^.

Moreover, the *Arabidopsis* U1 snRNP also promotes the usage of proximal CPA sites in 3′-UTRs, which might cause later RNAPII release in U1 knockdown lines. The underlying mechanism by which the *Arabidopsis* U1 snRNP suppresses intronic CPA sites while promoting proximal CPA sites in 3′-UTRs remains to be investigated. An RNAi screen in mouse cells shows that the knockdown of CPAFs results in contrasting effects on mRNA length, suggesting that some CPAFs promote while others inhibit certain CPA sites^96^. For example, the knockdown of FIP1 increases 3′-UTR length while the knockdown of CFIm68 results in shorter 3′-UTRs^96^. Interestingly, CFIm68 has been shown to act as an activator of premature polyadenylation within introns, suggesting a different role from its effect on 3′-UTR length. It was proposed that the U1 snRNP might prevent CFIm68 from associating with proximal CPA sites, thereby influencing its activity^36^. We found that CFIm68 and FIP1 associate with the *Arabidopsis* U1 snRNP, which might suggest that several U1–CPAF complexes with distinct activities exist in *Arabidopsis*. Depending on the composition of these complexes and the position along the gene, U1 snRNP might suppress CPAF activities, while a U1 snRNP with distinct protein partners might enhance cleavage and polyadenylation at proximal sites in 3′-UTRs (Fig. 6e). Identification of factors responsible for the distinct modes of U1 action will be an interesting subject for future studies.

Alternative polyadenylation plays a pivotal role in gene expression control in plants, and several factors involved in APA have been described in plants^57,58,61,97–100^. The U1 snRNP has not yet been linked to APA in plants, but our findings that the U1 snRNP regulates telescripting and 3′-UTR length may have important implications for adaptive gene regulation in plants. Early reports suggest that CPA rarely occurs within introns^101,102^. However, the usage of intronic CPA sites to regulate gene expression in *Arabidopsis* has been described in several instances. Such APA might lead to non-functional RNAs, which can control the abundance of the canonical mRNA or might generate alternative mRNAs encoding alternative protein isoforms^103–108^. Modulation of U1 snRNP telescripting function to regulate APA might therefore add an important layer for gene expression in *Arabidopsis* as well as in crops. Whether certain conditions globally affect telescripting in plants, as reported in human cells under heat-shock conditions, remains to be elucidated^34^.

## Methods

### Plant material and growth conditions

All *A. thaliana* lines used in this study were of the Columbia (Col-0) background. Plants for leaf measurement and visual documentation were grown on soil under long-day conditions (16 h light/8 h dark) at 22 °C/20 °C. For seedlings grown on plates, seeds were first surface sterilized with 80% ethanol containing 0.05% Triton X-100. Afterwards, seeds were grown on half-strength Murashige and Skoog (MS) plates containing 0.8% phytoagar for 7 days (for all RNA-sequencing approaches) or 14 days (for ChIP or ChIRP) under continuous light conditions at 22 °C.

For the construction of artificial microRNAs against *U1-70K* and *U1-C*, oligonucleotides (Supplementary Table 5) were derived from Web MicroRNA Designer (WMD3; http://wmd3.weigelworld.org/cgi-bin/webapp.cgi?page=Home;project=stdwmd). The PCR products were amplified using Phusion High Fidelity DNA polymerase (NEB) and a pRS300 plasmid containing the miR319a precursor as the template^109,110^. The engineered artificial microRNAs were subcloned into the pCR8/GW/TOPO vector (Thermo Fisher) and transferred into a Gateway Cloning system pGWB602 (ref. ^111^) using Gateway LR Clonase II Enzyme Mix (Thermo Fisher). The resulting plasmids were transformed into the *Agrobacterium tumefaciens* strain GV3101 and introduced into *A. thaliana* Col-0 plants by floral dipping^112^.

### RNA extractions, RT–qPCR and Illumina library preparation

Total RNA was extracted using Direct-zol RNA Miniprep (Zymo Research) according to manufacturer instructions. For the validation of the alternative splicing defects, 1–2 µg of RNA were treated with DNase I (Thermo Fisher), and the complementary DNA was prepared using the RevertAid First Strand cDNA Synthesis kit (Thermo Fisher) using 100 µM oligodT. RT–PCR was performed using the Dream*taq* DNA polymerase (Thermo Fisher) and run on 2% agarose gel. For the RT–qPCR experiments, we used Maxima SYBR Green (Thermo Fisher) in a Bio-Rad CFX-384 system and calculated the relative expression using the 2^−ΔΔCt^ with the *PP2A* gene as control. All the oligonucleotides are listed in Supplementary Table 5.

For the RNA-sequencing experiments, 5 µg of RNA was treated with DNase I (Thermo Fisher) and cleaned up using the RNA Clean and Concentrator-5 (Zymo Research). Poly(A) mRNA was isolated using the NEBNext Poly(A) mRNA Magnetic Isolation Module (New England Biolabs). Afterwards, the cDNA libraries were prepared using the NEBNext Ultra Directional RNA Library Prep kit for Illumina (New England Biolabs). The resulting libraries were measured using the Qubit dsDNA High Sensitivity Assay kit (Thermo Fisher) and size distribution was determined using the Agilent High Sensitivity DNA kit. Libraries were pooled together for paired-end sequencing on an Illumina Hi-Seq 3000 sytem. For 3′-end RNA sequencing, DNase-treated RNA was sent to Lexogen for library construction using the Quantseq 3′ mRNA-seq Library Prep kit REV.

### Differential gene expression and alternative splicing analysis

Paired-end reads were trimmed using Trim Galore (v.0.6.7; https://github.com/FelixKrueger/TrimGalore) with Cutadapt^113^ (v.3.4) and filtered by aligning all reads to the pre-transfer (t)RNA and ribosomal (r)RNA transcripts of *A. thaliana*. For this purpose, the latest transcriptome (ATRTD3) was queried for pre-tRNA and rRNA transcripts using the functional descriptions provided by Araport11 (refs. ^114,115^). The trimmed reads were then aligned to the custom pre-tRNA/rRNA reference using HISAT2 (v.2.2.1)^116^. Reads that did not align to any pre-tRNA or rRNA were used for downstream analysis. Quality control was performed before and after trimming and filtering with fastQC (v.0.11.9) (https://www.bioinformatics.babraham.ac.uk/projects/fastqc/) and summarized with multiQC (v.1.13)^117^. Filtered and trimmed reads were quantified at transcript level with salmon (v.1.9.0) using ATRTD3 (refs. ^115,118^). Quantified transcript reads were summarized to gene level and imported to R (v.4.2.2; https://www.R-project.org/) using tximport (v.1.26.1)^119^. After clustering analysis using PCA and hierarchical clustering combined with a heat map, it was evident that *amiR-u1-c* replicate 3 strongly differed from the rest of the samples. It was therefore excluded from further analysis. Differentially expressed genes (*P* < 0.05) were called using the R package DESeq2 (v.1.38.3)^120^. Additional packages used for the analysis and visualization are ggrepel (v.0.9.5; https://github.com/slowkow/ggrepel), ggplot2 (v.3.5.1)^121^ and dplyr (v.1.1.4; https://github.com/tidyverse/dplyr). For a full session report and additional quality control plots, refer to the Jupyter Notebook provided in the GitHub repository at https://github.com/WeberJoachim/Mangilet_et_al_2023 (ref. ^122^) within the subfolder ‘shortread_RNAseq’.

For the analysis of differentially spliced transcripts, the filtered and trimmed reads were mapped to the *Arabidopsis* genome (v.TAIR10)^123^ with HISAT2 (v.2.2.1). The resulting alignments were converted to BAM format, sorted and indexed using SAMtools (v.1.9)^124^. Differentially spliced transcripts were identified from indexed and sorted BAM files with rMATS (v.4.1.2). An additional software used in this analysis is seqkit (v.2.3.1)^125^. All workflows and specific parameters used in this analysis are available on GitHub at https://github.com/WeberJoachim/Mangilet_et_al_2023 (ref. ^122^) within the subfolder ‘shortread_RNAseq’.

### 3′-end mRNA sequencing analysis

Alternative CPA events were identified from the 3′-end mRNA sequencing reads using the apa toolkit within the expressRNA framework^74^. Data tables for APA were downloaded from expressRNA and summarized, overlap tested and plotted using R. For visualization, the reads were trimmed using Trim Galore and filtered by aligning to a pre-tRNA/rRNA reference using HISAT2. The filtered and trimmed reads were aligned to TAIR10 using HISAT2, and resulting alignment files were converted to BAM format, sorted and indexed using SAMtools. Parallel to the above-mentioned short-read RNA sequencing, the sample amiR-u1-c replicate 3 differed from the rest of the samples. This is in line with the fact that the RNA for 3′-end mRNA sequencing and RNA sequencing originate from the same biosample (SAMEA114383847). It was therefore excluded from the analysis. Pileups in the BedGraph format were generated from the sorted and indexed BAM files using deepTools (v.3.5.2)^126^ and merged using UCSC WiggleTools (v.1.2.8)^127^. Merged BedGraph files were further used for visualization.

### Nanopore direct RNA sequencing

Total RNA was isolated using RNAzol RT (Sigma-Aldrich, R4533) from three biological replicates of WT, *amiR-u1-70k* and *amiR-u1-c* seedlings according to manufacturer instructions and quantified using a NanoDrop ND-1000 spectrophotometer. We isolated poly(A) RNA using the Ambion Poly(A)Purist MAG K kit (Thermo Fisher, AM1922) according to manufacturer instructions. Quantity and quality of total and poly(A)-selected RNA were determined using the Qubit RNA HS assay and 2100 Agilent Bioanalyzer with the Agilent RNA 6000 Pico kit. For direct RNA-seq library preparation, the SQK-RNA002 kit (Oxford Nanopore Technologies) was used together with NEBNext Quick Ligation Reaction buffer (NEB B6058), T4 DNA Ligase 2 million U ml^−1^ (NEB, M0202), SuperScript III reverse transcriptase (Thermo Fisher, 18080044) and Agencourt RNAClean XP beads according to manufacturer instructions. Qubit 1x dsDNA HS assay was used to quantify 1 µl of the library, and the remainder was loaded on a primed PromethION flow cell (FLO-PRO002 R9) and run on a PromethION sequencer. The resulting fast5 files were basecalled using Cuda (v.12.1.0; https://developer.nvidia.com/cuda-12-1-0-download-archive) and Guppy (v.6.2.1) with the statistical model ‘rna_r9.4.1_70bps_hac_prom.cfg’. Initial quality analysis was performed using FastQC and summarized with multiQC. Basecalled reads were aligned against the genome (TAIR10) using minimap2 (v.2.24)^128^. SAM files were converted to BAM, sorted and indexed using SAMtools (v.1.17). Because of variation in library sizes ranging from 0.1 to 2.6 million reads within replicates, the alignments from all three biological replicates were collapsed using SAMtools to perform the qualitative analysis depicted in Figs. 4d, 5e and 6a,c.

### Comprehensive identification of RNA-binding proteins

The original protocol was adapted from ref. ^37^ with some minor modifications. Nine grams of 14-day-old *A. thaliana* Col-0 seedlings were harvested and crosslinked with 3% formaldehyde for 15 min under a vacuum chamber at 85 kPa. Vacuum infiltration was repeated once more to ensure proper crosslinking. The crosslinking reaction was then quenched by adding 4 ml of 1.25 M glycine for 5 min in the vacuum. Crosslinked seedlings were then washed three times with distilled water, dried on blotting paper and stored at −80 °C. To isolate the nuclei, frozen materials were grounded with liquid nitrogen and resuspended in HONDA buffer (400 mM sucrose, 1.25% Ficoll, 2.5% dextran, 25 mM HEPES-KOH pH 7.4, 10 mM MgCl_2_, 0.5% Triton X-100, 1 mM phenylmethylsulfonyl fluoride (PMSF), Complete Protease Inhibitor Cocktail EDTA-free (Roche) and 10 mM dithiothreitol (DTT)). The homogenate was passed through two layers of Miracloth and centrifuged at 1,500*g* for 15 min at 4 °C. The pellet was carefully washed five times with HONDA buffer until most of the green material was removed. For washing, the sample was centrifuged at 1,500*g* for 5 min at 4 °C. A final wash with M3 buffer (10 mM sodium phosphate pH 7.0, 100 mM NaCl, 10 mM DTT and 1X protease inhibitor) was done before the pellet was resuspended in sonic buffer (10 mM sodium phosphate pH 7.0, 100 mM NaCl, 0.5% sarkosyl, 10 mM EDTA, 1X Complete cocktail, 1 mM Pefabloc SC). Chromatin shearing was done using the Covaris S220 under the following conditions: 20% duty cycle, 140 peak intensity, 200 cycles per burst and a total of 3 min of cycle time. The samples were centrifuged at 16,000*g* for 5 min at 4 °C. The supernatant containing the chromatin was then transferred into a DNA LoBind tube (Eppendorf), flash frozen in liquid nitrogen and stored at −80 °C. The chromatin was thawed at room temperature together with the probes for the U1 snRNA and the control RNA (Supplementary Table 5). Fifty microliters of chromatin served as the protein input. Two milliliters of hybridization buffer (750 mM NaCl, 50 mM Tris-HCl pH 7.0, 1 mM EDTA, 1% SDS, 15% formamide, 1x protease inhibitor, 1x PMSF, 1x RiboLock (40 U µl^−1^; Thermo Fisher), plant-specific protease inhibitor (Sigma)) was added to 1 ml chromatin. After adding 100 pmol probe per ml chromatin, the samples were gently rotated end-to-end at 37 °C for 4 h for hybridization. With 2 h remaining for the hybridization, 100 µl of Dynabeads MyOne Streptavidin C1 (Thermo Fisher) were prepared by removing the storage buffer and washing three times with 1 ml of unsupplemented nuclear lysis buffer (50 mM Tris-HCl, 10 mM EDTA, 1% SDS) using a magnetic stand. When the hybridization was finished, 100 µl of the washed beads were added to the mixture and the mixture was incubated for an additional 30 min. During this incubation, the wash buffer (2x SSC, 0.5% SDS) was prepared and pre-warmed at 37 °C before use. When the bead binding was completed, the mixture was briefly centrifuged and the beads were separated from the mixture for 2 min in a magnetic stand. One microliter of the wash buffer was used to wash the beads, followed by gentle rotation at 37 °C for 5 min in a hybridization oven. The washing step was repeated four times, for a total of five washes. For the last wash, all buffer was removed. For the preparation for the mass spectrometry analysis, the beads were washed three times in 20 mM sodium bicarbonate buffer.

### Protein on beads digestion

All steps for protein digestion were performed at room temperature as described previously^129^. Briefly, beads were resuspended in denaturation buffer (6 M urea, 2 M thiourea, 10 mM Tris buffer, pH 8.0), and proteins were reduced and subsequently alkylated by incubation in 1 mM DTT for 1 h, followed by addition of 5.5 mM iodacetamide for another hour in the dark. Proteins were pre-digested with LysC for 3 h at pH 8.0. Beads were then diluted in four volumes 20 mM ammonium bicarbonate buffer and proteins digested with 2 µg trypsin per estimated 100 µg protein at pH 8.0 overnight. Acidified peptides were desalted with C18 stage tips as described previously^130^.

### Mass spectrometry

LC–MS/MS analyses of eluted samples were performed on an Easy nano-LC (Thermo Fisher) coupled to an LTQ Orbitrap XL mass spectrometer (Thermo Fisher) as described in ref. ^131^. The peptide mixtures were injected onto the column in HPLC solvent A (0.1 % formic acid) at a flow rate of 500 nl min^−1^ and subsequently eluted with a 49 min segmented gradient of 10-33-50-90% of HPLC solvent B (80% acetonitrile in 0.1% formic acid) at a flow rate of 200 nl min^−1^. The 15 most intense precursor ions were sequentially fragmented in each scan cycle using collision-induced dissociation. In all measurements, sequenced precursor masses were excluded from further selection for 30 s. The target values were 5,000 charges for MS/MS fragmentation and 10^6^ charges for the MS scan. Due to high contamination of polymers in the samples, it was decided to further purify the samples via PHOENIX Peptide Clean-up kit (PreOmics) according to the user manual. Final measurements were performed after PHOENIX kit purification as described above.

### Mass spectrometry data processing

The MS data of all runs together were processed with MaxQuant software suite (v.1.5.2.8)^132^. A database search was performed using the Andromeda search engine, which is integrated into MaxQuant^133^. MS/MS spectra were searched against a target-decoy Uniprot database for *A. thaliana* downloaded on 13 February 2019, consisting of 91,457 protein entries from *A. thaliana* and 245 commonly observed contaminants. In a database search, full specificity was required for trypsin. Up to two missed cleavages were allowed. Carbamidomethylation of cysteine was set as a fixed modification, whereas oxidation of methionine and acetylation of protein N terminus were set as variable modifications. Initial mass tolerance was set to 4.5 parts per million for precursor ions and 0.5 Da for fragment ions. Peptide, protein and modification site identifications were reported at a false discovery rate (FDR) of 0.01, estimated by the target/decoy approach^134^. Match between runs was enabled for samples within one group, so for U1, U2 and control samples separately. Intensity-based absolute quantification (iBAQ) and label-free quantification settings were enabled. MaxQuant data were analysed using msVolcano^135^ for the detection of significantly enriched proteins using the following parameters: FDR = 0.04, curvature = 0.75, minimum fold change = 0; or FDR = 0.05, curvature = 2.5; minimum fold change = 0 for U1-IP–MS and U2-IP–MS, respectively.

### Co-immunoprecipitation

For the expression of HA-, RFP- or YFP-tagged proteins, the coding sequence of each protein was PCR amplified and subcloned into the vector pCR8/GW/TOPO (Invitrogen). To generate binary plasmids, the entry vectors were recombined using Gateway LR Clonase II (Thermo Fisher) with either pGWB642 for the expression of YFP-tagged fusion proteins, pGWB515 for the expression of HA-tagged fusion proteins or pGWB654 for the expression of RFP fusion proteins^111^. Binary plasmids were transformed into *Agrobacterium tumefaciens* (strain GV3101). Proteins were expressed by *Agrobacterium*-mediated transient expression in *Nicotiana benthamiana*. For this, *Agrobacterium* was grown overnight at 28 °C and cultures were pelleted by centrifugation. The pellets were resuspended in infiltration media (10 mM MgCl_2_, 10 mM MES-KOH, pH 5.6 and 100 µM acetosyringone) and the optical density (OD)_600_ was adjusted to 0.5. After being incubated for 3 h at 22 °C with light agitation, one or two leaves per *N. benthamiana* plant were infiltrated with above-mentioned infiltration media. After 3 days, transformed tobacco leaves were snap frozen, grounded to a fine powder and resuspended in protein lysis buffer (50 mM Tris-HCl pH 7.5, 150 mM NaCl, 10% glycerol, 0.5% Triton X-100, 0.5% Nonidet P 40 Substitute, 1 mM PMSF, 2 mM DTT, 50 µM MG132, plant-specific protease inhibitor (Sigma-Aldrich, P9599) and Complete Protease Inhibitor Cocktail EDTA-free (Roche)). After centrifugation at 13,000*g* for 10 min at 4 °C, the supernatant was used for immunoprecipitation. For each immunoprecipitation, 20 µl of RFP-trap beads (Chromotek) were equilibrated by washing three times with wash buffer (50 mM Tris-HCl pH 7.5, 150 mM NaCl, 10% glycerol). The protein samples were added to the equilibrated beads and incubated for 1 h on a rotating wheel at 4 °C. For the experiments shown in Fig. 2d,e, RNase A at a final concentration of 10 µg ml^−1^ was added to the IP samples. The input samples were incubated together with the IP samples. After incubation, the beads were washed three times with wash buffer before incubation in Laemmli buffer at 80 °C for 10 min. The isolated proteins were resolved by SDS–PAGE, blotted to nitrocellulose membranes and incubated with antibodies specific for GFP (Chromotek, 3h9; 1:1,000 dilution), RFP (Chromotek, 6g6; 1:2,000 dilution), HA (Agrisera, AS12 2200; 1:3,000 dilution) or MYC (Sigma, C3956; 1:2,000 dilution). HRP-conjugated secondary antibodies (anti-rat AS10 1115, Agrisera, 1:2,500 dilution; anti-rabbit AS09 602, Agrisera, 1:25,000 dilution; and anti-mouse AS10 1115, Agrisera, 1:5,000 dilution) and the Western Bright Chemiluminescence Substrate Sirius (Biozym) were used for protein detection.

### Chromatin immunoprecipitation

The method was adapted from ref. ^136^. Three grams of 14-day-old *Arabidopsis* seedlings were collected and fixed with 40 ml 1% formaldehyde in MQ buffer (10 mM sodium phosphate pH 7.0, 50 mM NaCl) for 10 min under a vacuum chamber at 85 kPa. Vacuum infiltration was repeated once more to ensure proper crosslinking. The crosslinking reaction was then quenched by adding 4 ml of 1.25 M glycine for 5 min in the vacuum. Crosslinked seedlings were then washed three times with distilled water, dried on paper and stored at −80 °C. To isolate the nuclei, frozen materials were grounded with liquid nitrogen and resuspended in HONDA buffer (400 mM sucrose, 1.25% Ficoll, 2.5% dextran, 25 mM HEPES-KOH pH 7.5, 10 mM MgCl_2_, 0.5% Triton X-100, 1 mM PMSF, Complete Protease Inhibitor Cocktail EDTA-free (Roche) and 10 mM DTT). The resuspended plant materials were filtered with 2 layers of Miracloth and transferred into a new 50 ml tube. The homogenate was centrifuged at 1,500*g* for 15 min at 4 °C. The pellet was carefully washed five times with HONDA buffer until most of the green material was removed. For washing, the sample was centrifuged at 1,500*g* for 5 min at 4 °C. A final wash with M3 buffer (10 mM sodium phosphate pH 7.0, 100 mM NaCl, 10 mM DTT and 1X proteinase inhibitor) was done before the pellet was resuspended in sonic buffer (10 mM sodium phosphate pH 7.0, 100 mM NaCl, 0.5% sarkosyl, 10 mM EDTA, 1X Complete cocktail, 1 mM PEFA). Chromatin shearing was done using a focused ultrasonicator (Covaris S220) under the following conditions: 20% duty cycle, 140 peak intensity, 200 cycles per burst and a total of 3 min of cycle time. The samples were centrifuged at 16,000*g* for 5 min at 4 °C. The supernatant was then transferred into a DNA LoBind tube.

For the immunoprecipitation experiment, 700 µl of the solubilized chromatin was used and 140 µl of the input. IP buffer (50 mM HEPES pH 7.4, 150 mM KCl, 5 mM MgCl_2_, 0.01 mM ZnSO_4_, 1% Triton X-100, 0.05% SDS) was then added to the IP and input. The antibody against RNAPII CTD (Abcam, ab817) was added to the IP and incubated overnight on a rotating wheel at 4 °C. The following day, 40 µl of Protein A/G agarose beads (Santa Cruz Biotechnology, sc2001) were added to the IP and incubated for 6 h in a rotating wheel at 4 °C. After incubation, the beads were pelleted by centrifugation and washed five times with 1 ml of IP buffer on a rotating wheel and centrifuged after each wash. Protein-associated DNA was eluted with 120 µl of cold acidic glycine buffer pH 2.8 (100 mM glycine, 500 mM NaCl, 0.05% Tween-20, HCl). The supernatant was transferred to a tube containing 150 µl of Tris pH 9.0. This elution with glycine was repeated twice and each elution was transferred into the same tube. RNase A was added and incubated at 37 °C for 15 min. To denature the proteins, 1.5 µl of Proteinase K was added and the mixture was incubated overnight at 37 °C. A second aliquot of Proteinase K was added to the samples and the mixture was incubated at 65 °C for 6 h to reverse the crosslinking. DNA was then purified using MinElute (Qiagen) according to manufacturer instructions with minor modifications. The IP samples were divided into two samples and three volumes of ERC buffer were added to each sample. The pH was adjusted using 3 M sodium acetate. The mixture was then added to the spin column, washed with the PE buffer and eluted with 35 µl EB buffer. ChIP DNA libraries were prepared using the NEBNext Ultra II DNA Library Prep kit for Illumina (New England Biolabs) according to manufacturer instructions. The libraries were prepared without size selection. Multiplexing was done using the NEBNext Multiplex Oligos for Illumina (Set 1, 2, 3, 4). The concentration of the libraries was determined using the Qubit dsDNA HS Assay kit (Thermo Fisher) and size distribution was measured using the Agilent High Sensitivity DNA kit (Agilent). Libraries were pooled together and paired-end sequencing was performed on an Illumina Hi-Seq 2000 system.

### Chromatin immunoprecipitation DNA-sequencing analysis

Paired-end reads from ChIP-seq were trimmed using Trim Galore. Trimmed reads were then aligned to the *Arabidopsis* genome (v.TAIR10) with HISAT2 using the ‘–no-splice-alignment’ option. Mapped reads were further analysed with MACS2 (v.2.9.1)^137^. Therefore, the IGG control pileups were subtracted from the treatment and input control pileups. The resulting pileups (BedGraphs) were compared using fold enrichment between IGG-corrected treatment and input. Quality control of pileups was performed by converting BedGraphs to bigWig files and subsequent multibigwigsummary and plotCorrelation using deepTools (v.3.5.2)^126^. During quality control of the samples by cluster analysis, it was discovered that replicate 1 of *amiR-u1-c* behaved differently from all other samples and was thus discarded in the downstream analysis. Metaplots were assembled by merging the bigWig files and then plotting them using deepTools plotProfile.

### SmallRNA sequencing

Total RNA was isolated using RNAzol RT (Sigma-Aldrich, R4533) from three biological replicates of Col-0, *amiR-u1-c* and *amiR-u1-70k* and quantified using a NanoDrop ND-1000 spectrophotometer. Library preparation and sequencing (SE50) was done by Novogene using an Illumina Novaseq6000 system.

### MicroRNA analysis

SmallRNA raw sequencing data were processed using nf-core^138^/smrnaseq (10.5281/zenodo.3456879, v.2.2.4) (https://nf-co.re/smrnaseq/2.2.4), a standardized publicly available Nextflow pipeline for small RNA-seq analyses. Briefly, quality control and visualization of raw sequence reads was done using FastQC (v.0.12.1) and multiQC (v.1.19). Adapter trimming and base quality filtering was performed using Fastp (v.0.23.4)^139^. Bowtie (v.1.3.1)^140^ was used to align the resulting reads against the *A. thaliana* miRNA reference from the miRBase database (https://mirbase.org/)^141^. Alignment processing and feature counting was performed using samtools (v.1.14). All analyses were done using default parameters for all the tools in the pipeline. Counts normalization and differential expression analysis were performed using DESeq2 (v.1.40.2). EnhancedVolcano (https://github.com/kevinblighe/EnhancedVolcano, v.1.18.0) was used for visualizing significantly (FDR ≤ 0.05 and |Fold change| ≥ 1) upregulated and downregulated miRNAs between genotypes.

### Gene Ontology term analysis

To test for overrepresentation of functions, processes or compartments in which a set of genes might be active, the PANTHER database was queried (Gene Ontology, 10.5281/zenodo.10536401). The gene identifiers were entered manually and compared to all *Arabidopsis* genes in the database. Fisher test was performed for significance testing and FDR was used to correct for the multiple-testing problem. Gene Ontology (GO) terms that were overrepresented within GO-molecular-function, GO-cellular-component or GO-biological-process were filtered using FDR < 0.05

### Data visualization

For visualizing all sequencing reads, we created a fork of the long-read visualization framework from FLEP-seq^142^ and added the functionality to plot BedGraph files. The code can be found in the Jupyter Notebook in the GitHub repository of this study or as a standalone repository on GitHub at https://github.com/WeberJoachim/Viz_bdg_and_nanopore_bam.

### Bioinformatic analysis

All custom analysis pipelines were implemented using Nextflow (v.23.10.0)^104^, utilizing containerization with Singularity Community Edition (v.3.11.4-1.el8)^105^. Singularity images were pulled from Galaxy Project^106^. Computational resources were provided by the HPC cluster CARL located at the University of Oldenburg (Germany) and funded by the DFG through its Major Research Instrumentation Programme (INST 184/157-1 FUGG) and the Ministry of Science and Culture (MWK) of the Lower Saxony State. CARL was replaced by the HPC cluster ROSA (INST 184/225-1 FUGG) during the research process.

### Reporting summary

Further information on research design is available in the Nature Portfolio Reporting Summary linked to this article.

## Supplementary information

Reporting Summary

Supplementary Tables 1–5 Supplementary Table 1. List of proteins that are significantly enriched in U1-IP-MS. Table 2. List of proteins that are significantly enriched in U2-IP-MS. Table 3. List of proteins significantly enriched by only U1-IP-MS or U2-IP-MS. Table 4. Statistical overrepresentation test of GO terms of U1 snRNA-associated proteins using the PANTHER database. Table 5. List of oligonucleotides used in the study.

Supplementary Data 1 Proteins identified by mass spectrometry after affinity purification using U1-, U2- and *lacZ*-RNA-specific antisense oligonucleotides.

Supplementary Data 2 Enrichment analysis of biological processes, functions and protein domains among U1 snRNA-associated proteins.

Supplementary Data 3 Differentially expressed genes in *amiR-u1-70k* and *amiR-u1-c*, respectively, compared with wild-type plants.

Supplementary Data 4 Overlap between alternatively spliced RNAs in *amiR-u1-70k* and *amiR-u1-c*, respectively, compared with WT.

Supplementary Data 5 Overlap between alternatively spliced and differentially expressed genes in *amiR-u1-70k* and *amiR-u1-c* plants.

Supplementary Data 6 Alternative polyadenylation events detected in WT, *amiR-u1-70k* and *amiR-u1-c* plants.

Supplementary Data 7 List of expression and splicing changes among known CPAFs in *amiR-u1-70k* and *amiR-u1-c* plants.

## Source data

Source Data Fig. 2 Unprocessed western blots.

Source Data Extended Data Fig. 1 Unprocessed western blots.

## Data Availability

All raw datasets, along with metadata files, are publicly available at ENA or PRIDE under the accession numbers PRJEB65251 (for RNA and DNA sequencing) and PXD045484 (for proteomic analyses). The *Arabidopsis* reference genome was obtained from TAIR (https://www.arabidopsis.org). *Arabidopsis* reference transcriptomes were sourced from https://ics.hutton.ac.uk/atRTD/RTD3/ (for AtRTD3) and https://phytozome-next.jgi.doe.gov/info/Athaliana_Araport11 (for ARAPORT11). *MiRNA* annotations were downloaded from miRBase (https://www.mirbase.org/browse/results/?organism=ath). Protein information was derived from UniProt (https://www.uniprot.org/), Panther (10.5281/zenodo.10536401) and STRING (https://string-db.org/). Source data are provided with this paper.

## Code availability

All analysis pipelines and parameters applied are accessible on GitHub at https://github.com/WeberJoachim/Mangilet_et_al_2023 (ref. ^122^).
