# Supplementary material for: The Arabidopsis U1 snRNP regulates mRNA 3′-end processing
Source: Nat Plants. 2024 Sep 23;10(10):1514–31. doi: 10.1038/s41477-024-01796-8 (PMC11489095; doi:10.1038/s41477-024-01796-8)
Supplement: Supplementary file 1 — Reporting Summary [file 41477_2024_1796_MOESM1_ESM.pdf]

nature portfolio

Corresponding author(s): Sascha Laubinger
Last updated by author(s): Aug 19, 2024

Reporting Summary

Nature Portfolio wishes to improve the reproducibility of the work that we publish. This form provides structure for consistency and transparency in reporting. For further information on Nature Portfolio policies, see our Editorial Policies and the Editorial Policy Checklist.

Statistics

For all statistical analyses, confirm that the following items are present in the figure legend, table legend, main text, or Methods section.

n/a	Confirmed	
☐	☒	The exact sample size (n) for each experimental group/condition, given as a discrete number and unit of measurement
☐	☒	A statement on whether measurements were taken from distinct samples or whether the same sample was measured repeatedly
☐	☒	The statistical test(s) used AND whether they are one- or two-sided<br>*Only common tests should be described solely by name; describe more complex techniques in the Methods section.*
☒	☐	A description of all covariates tested
☐	☒	A description of any assumptions or corrections, such as tests of normality and adjustment for multiple comparisons
☐	☒	A full description of the statistical parameters including central tendency (e.g. means) or other basic estimates (e.g. regression coefficient) AND variation (e.g. standard deviation) or associated estimates of uncertainty (e.g. confidence intervals)
☐	☒	For null hypothesis testing, the test statistic (e.g. F, t, r) with confidence intervals, effect sizes, degrees of freedom and P value noted<br>*Give P values as exact values whenever suitable.*
☒	☐	For Bayesian analysis, information on the choice of priors and Markov chain Monte Carlo settings
☒	☐	For hierarchical and complex designs, identification of the appropriate level for tests and full reporting of outcomes
☐	☒	Estimates of effect sizes (e.g. Cohen's d, Pearson's r), indicating how they were calculated

Our web collection on statistics for biologists contains articles on many of the points above.

Software and code

Policy information about availability of computer code

Data collection	No software was used
Data analysis	General Illumina read processing<br>Trimming of paired end reads: Trim Galore (version 0.6.7) with Cutadapt (version 3.4)<br>Read aligning: HISAT2 (version 2.2.1).<br>Quality control: fastQC (version 0.11.9) and multiQC (version 1.13).<br><br>Differential gene expression analysis:<br><br>Read quantification: salmon (version 1.9.0) using ATRTD3 as reference transcriptome.<br>Quantified transcript level reads were summarized to gene level and imported to R (version 4.2.2) using tximport (version 1.26.1)).<br>Calling of differentially expressed genes (p < 0.05): DESeq2 (version 1.38.3).<br><br>GO-Term analysis: PANTHER database doi: 10.5281/zenodo.10536401<br><br>MicroRNA analysis:<br>Quality control: FastQC (version 0.12.1) and multiQC (version 1.19).<br>Adapter trimming and base quality filtering: Fastp (version 0.23.4)138.<br>Alignment: Bowtie (version 1.3.1)139 using the Arabidopsis thaliana miRNA reference from miRBase database (https://mirbase.org/)140.<br>Alignment processing and feature counting: samtools (version 1.14) using default parameters for all the tools in the pipeline.<br>Counts normalization and differential expression analysis: DESeq2 (version 1.40.2).

Visualization: EnhancedVolcano (https://github.com/kevinblighe/EnhancedVolcano., version 1.18.0)

Differential splicing analysis:
Read mapping: HISAT2 (version 2.2.1) with TAIR10 as reference genome. The alignments were converted to BAM format, sorted and indexed using SAMtools (version 1.9) and seqkit (version 2.3.1). Differentially spliced transcripts were identified from indexed and sorted BAM files with rMATS (version 4.1.2).

3'-end mRNA sequencing analysis:
Read mapping: HISAT2 (version 2.2.1) with TAIR10 as reference genome
3'-end mRNA sequencing analysis: apa toolkit within expressRNA framework.

Nanopore direct RNA-sequencing
ONT sequence raw data was basecalled using Guppy (v6.2.1).
Initial quality checks by FastQC (v0.12.0).
Sequence alignment performed with minimap2 (v2.24).
Format conversion using samtools (v1.17).

ChIP-seq analysis
Read alignment: HISAT2 using the "--no-splice-alignment" option against the TAIR10 genome
ChIP analysis: MACS2 (version 2.9.1.). The resulting pileups (BedGraphs) were then compared using fold enrichment between IgG controls and input.
Quality control: The pileups were converted from BedGraphs to bigWig files and analyzed using multibigwigsummary and plotCorrelation in deepTools (version 3.5.2)

Additional packages used for the analysis and visualization: ggrepel (version 0.9.3, https://github.com/slowkow/ggrepel), ggplot2 (version 3.4.2) and dplyr (version 1.1.2)

Mass spectrometry data processing
MS data processing: MaxQuant software suite v.1.5.2.8.
MaxQuant data visualization: msVolcano, DOI: 10.1002.pmic.201600167, online version accessed Oct. 25, 2021

RNA-seq and ChIP-seq data vizualization: IGV, version 2.16.0, downloaded Jan 25, 2023

All costum codes and parameter are available under https://github.com/WeberJoachim/Mangilet_et_al_2023

For manuscripts utilizing custom algorithms or software that are central to the research but not yet described in published literature, software must be made available to editors and reviewers. We strongly encourage code deposition in a community repository (e.g. GitHub). See the Nature Portfolio guidelines for submitting code & software for further information.

Data

Policy information about availability of data

All manuscripts must include a data availability statement. This statement should provide the following information, where applicable:

- Accession codes, unique identifiers, or web links for publicly available datasets
- A description of any restrictions on data availability
- For clinical datasets or third party data, please ensure that the statement adheres to our policy

All raw data sets, along with metadata files, are publicly available at ENA or PRIDE under the accession numbers PRJEB65251 (for RNA and DNA sequencing) or PXD045484 (for proteomic analyses). The Arabidopsis reference genome was obtained from TAIR (https://www.arabidopsis.org). Arabidopsis reference transcriptomes were sourced from https://ics.hutton.ac.uk/atRTD/RTD3/ (for AtRTD3) and https://phytozome-next.jgi.doe.gov/info/Athaliana_Araport11 (for ARAPORT11). MiRNA annotations were downloaded from miRBase (https://www.mirbase.org/browse/results/?organism=ath). Protein information was derived from UniProt (https://www.uniprot.org/), Panther (10.5281/zenodo.10536401) and STRING (https://string-db.org/).

Research involving human participants, their data, or biological material

Policy information about studies with human participants or human data. See also policy information about sex, gender (identity/presentation), and sexual orientation and race, ethnicity and racism.

Reporting on sex and gender	does not apply
Reporting on race, ethnicity, or other socially relevant groupings	does not apply
Population characteristics	does not apply
Recruitment	does not apply
Ethics oversight	does not apply

Note that full information on the approval of the study protocol must also be provided in the manuscript.

Field-specific reporting

Please select the one below that is the best fit for your research. If you are not sure, read the appropriate sections before making your selection.

☒ Life sciences ☐ Behavioural & social sciences ☐ Ecological, evolutionary & environmental sciences

For a reference copy of the document with all sections, see nature.com/documents/nr-reporting-summary-flat.pdf

Life sciences study design

All studies must disclose on these points even when the disclosure is negative.

Sample size	For ChIRP-MS, RNA-seq and ChIP-seq experiments, we performed three biological replicates. These replicates consist of pooled Arabidopsis seedlings (100s for ChIRP-MS and ChIP-seq, or at least 15 seedlings for RNA-seq). Sampling (pooling of individual Arabidopsis seedlings) and replication (usually three biological replicates) resulted in robust and reproducible results in former experiments.
Data exclusions	Quality control measures revealed that one biological replicate of amiR-u1-c behave differently in RNA-seq and ChIP-seq, probably due to an unintentional contamination with WT seed. We there exclude this replicate from all analyses.
Replication	CHIRP, RNA-seq and ChIP-seq experiments were performed in three biological replicates. Co-Immunoprecipitation experiments were performed two or three times independently. The extact number of replication is stated in the figure legends. We put utmost care to perform orthogonal replication of all experiments. E.g. ChIRP experiments were confirmed by performing co-immunoprecipitation experiments. Splicing defects detected by RNA-seq were confirmed in independent biological replicates by RT-PCR and ONT-direct RNA-seq. Cleavage and polyadenylation defects detected by 3'-seq were confirmed in independent biological replicates by ONT-direct RNA-seq. All attempts at replication were successful.
Randomization	Plants was grown in growth cabinets in a randomized fashion. Plant material was collected from plates randomly placed in growth cabinents.
Blinding	Blinding was not possible because the knockdown lines we work with exhibited very distinctive phenotypes.

Reporting for specific materials, systems and methods

We require information from authors about some types of materials, experimental systems and methods used in many studies. Here, indicate whether each material, system or method listed is relevant to your study. If you are not sure if a list item applies to your research, read the appropriate section before selecting a response.

Materials & experimental systems

n/a	Involved in the study	
☐	☒	Antibodies
☒	☐	Eukaryotic cell lines
☒	☐	Palaeontology and archaeology
☒	☐	Animals and other organisms
☒	☐	Clinical data
☒	☐	Dual use research of concern
☐	☒	Plants

Methods

n/a	Involved in the study	
☐	☒	ChIP-seq
☒	☐	Flow cytometry
☒	☐	MRI-based neuroimaging

Antibodies

Antibodies used	For ChIP-seq: anti-RNAPII CTD (Abcam, ordering number ab817)<br>For protein blot analyses: anti-GFP (Chromotek, ordering number 3h9), anti-RFP (Chromotek, ordering number 6g6), anti-HA (Agrisera, ordering numberAS12 2200), anti-MYC (Sigma, ordering number C3956).<br>HRP-conjugated secondary antibodies: (all Agrisera: anti-rat, ordering number AS10 1115; anti-rabbit, ordering number AS09 602; and anti-mouse, ordering numberAS10 1115)
Validation	All antibodies were tested for specificity using Arabidopsis samples or protein extracts of infiltrated and non-infiltrated Nicotiana benthamiana plants. Manufacturers' validations can be found under the following links: https://www.ptglab.com/products/GFP-antibody-3H9.htm (for anti-GFP), https://www.ptglab.com/products/RFP-antibody-6G6.htm (for anti-RFP), https://www.agrisera.com/en/artiklar/ha-tag.html (for anti-HA), https://www.sigmaaldrich.com/DE/de/product/sigma/c3956?srsltid=AfmBOop9jSOWGklafmxqn5hAADHIP97jg4VHFV2k5pJ1NHT4ItJw3LYA (for anti-MYC), https://www.citeab.com/antibodies/752154-ab817-anti-rna-polymerase-ii-ctd-repeat-ysptsps-antib (for anti-RNAPII CTD).

Dual use research of concern

Policy information about dual use research of concern

Hazards

Could the accidental, deliberate or reckless misuse of agents or technologies generated in the work, or the application of information presented in the manuscript, pose a threat to:

No	Yes	
☒	☐	Public health
☒	☐	National security
☒	☐	Crops and/or livestock
☒	☐	Ecosystems
☒	☐	Any other significant area

Experiments of concern

Does the work involve any of these experiments of concern:

No	Yes	
☒	☐	Demonstrate how to render a vaccine ineffective
☒	☐	Confer resistance to therapeutically useful antibiotics or antiviral agents
☒	☐	Enhance the virulence of a pathogen or render a nonpathogen virulent
☒	☐	Increase transmissibility of a pathogen
☒	☐	Alter the host range of a pathogen
☒	☐	Enable evasion of diagnostic/detection modalities
☒	☐	Enable the weaponization of a biological agent or toxin
☒	☐	Any other potentially harmful combination of experiments and agents

Plants

Seed stocks	All plant transgenic plants used in this study have not been described before.
Novel plant genotypes	We generated artificial amiRNA lines against U1-70K and U1-C. We screened 40 independent primary transformats, which exhibit very similar altered phenotypes compared to WT. Experiments were performed with homozygous T4 or T5 plants.
Authentication	Plants were selected from single-insertion lines (controlled in T2 generation). The expression of of U1-70K and U1-C was tested by qRT-PCR. All experiments were always performed with amiR-u1-70k and amiR-u1-c lines. Because U1-70K and U1-C are subunit of the same protein complex, and we obtained very similar results with both transgenic lines, we consider the effect of the knockdown very reliable.

ChIP-seq

Data deposition

☒ Confirm that both raw and final processed data have been deposited in a public database such as GEO.

☒ Confirm that you have deposited or provided access to graph files (e.g. BED files) for the called peaks.

Data access links<br>*May remain private before publication.*	All ChIP-seq data sets are publicly available at ENA under the accession numbers PRJEB65251
Files in database submission	fastq and bam files for<br>WT, input, replicate 1<br>WT, input, replicate 2<br>WT, input, replicate 3<br>WT, IgG control, replicate 1<br>WT, IgG control, replicate 2<br>WT, IgG control, replicate 3<br>WT, IP anti-RNAPII CTD, replicate 1

WT, IP anti-RNAPII CTD, replicate 2
WT, IP anti-RNAPII CTD, replicate 3
amiR-u1-70k, input, replicate 1
amiR-u1-70k, input, replicate 2
amiR-u1-70k, input, replicate 3
amiR-u1-70k, IgG control, replicate 1
amiR-u1-70k, IgG control, replicate 2
amiR-u1-70k, IgG control, replicate 3
amiR-u1-70k, IP anti-RNAPII CTD, replicate 1
amiR-u1-70k, IP anti-RNAPII CTD, replicate 2
amiR-u1-70k, IP anti-RNAPII CTD, replicate 3
amiR-u1-c, input, replicate 1
amiR-u1-c, input, replicate 2
amiR-u1-c, input, replicate 3
amiR-u1-c, IgG control, replicate 1
amiR-u1-c, IgG control, replicate 2
amiR-u1-c, IgG control, replicate 3
amiR-u1-c, IP anti-RNAPII CTD, replicate 1
amiR-u1-c, IP anti-RNAPII CTD, replicate 2
amiR-u1-c, IP anti-RNAPII CTD, replicate 3

Genome browser session (e.g. UCSC)

we make bigwig files available under https://figshare.com/articles/dataset/Bigwigs_of_PolII_Chipseq_in_Col-0_amiR-u1c_and_amiR-u170k/26820682
These files can be uploaded into IGV (https://www.igv.org/) for convenient visualization of the ChIP-seq results

Methodology

Replicates

We performed three biological replicates

Sequencing depth

Sequencing resulted in between 21 and 35 million reads per replicate

Antibodies

anti-RNAPII CTD (Abcam, ordering number ab817)

Peak calling parameters

We did not perform any peak calling

Data quality

For quality control, the pileups were converted from BedGraphs to bigWig files and analyzed using multibigwigsummary and plotCorrelation in deepTools (version 3.5.2).

Software

Read alignment: HISAT2 using the "--no-splice-alignment" option against the TAIR10 genome
ChIP analysis: MACS2 (version 2.9.1.). The resulting pileups (BedGraphs) were then compared using fold enrichment between IgG controls and input.
