# Supplementary material for: The Arabidopsis U1 snRNP regulates mRNA 3′-end processing
Source: Nat Plants. 2024 Sep 23;10(10):1514–31. doi: 10.1038/s41477-024-01796-8 (PMC11489095; doi:10.1038/s41477-024-01796-8)

Unprocessed blots for Figure 2B

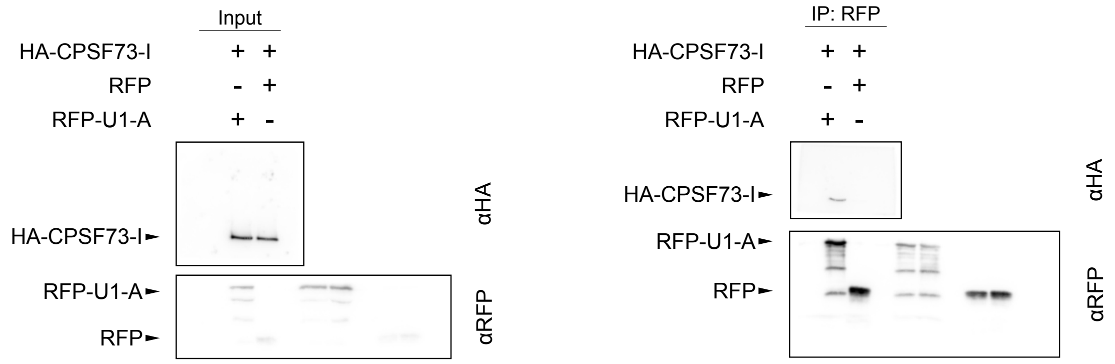

Unprocessed blots for Figure 2C

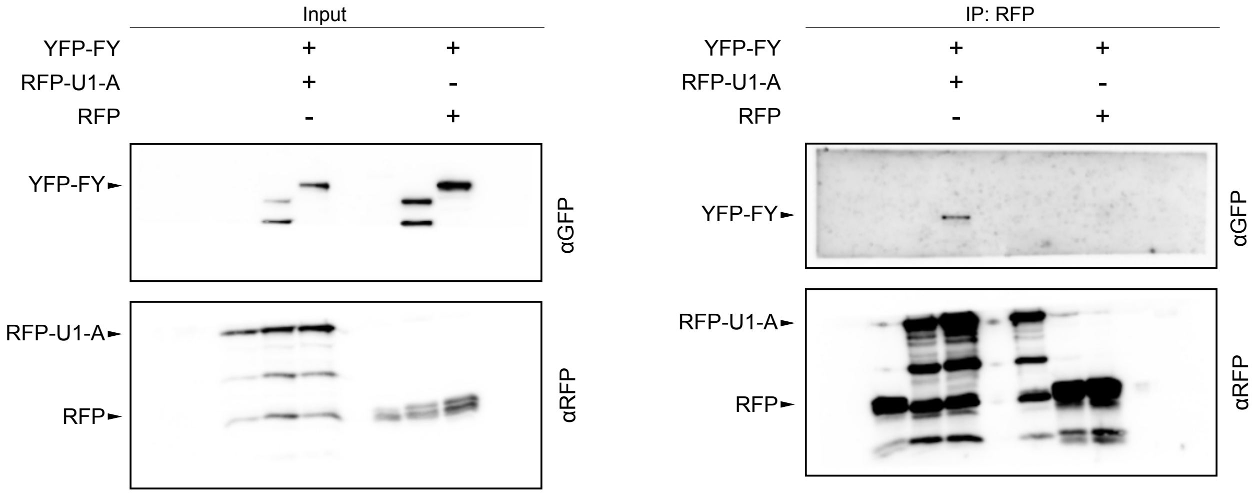

Unprocessed blots for Figure 2D

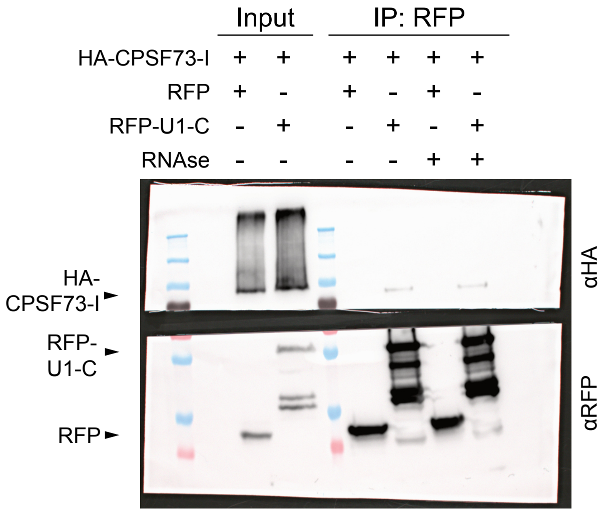

Unprocessed blots for Figure 2E

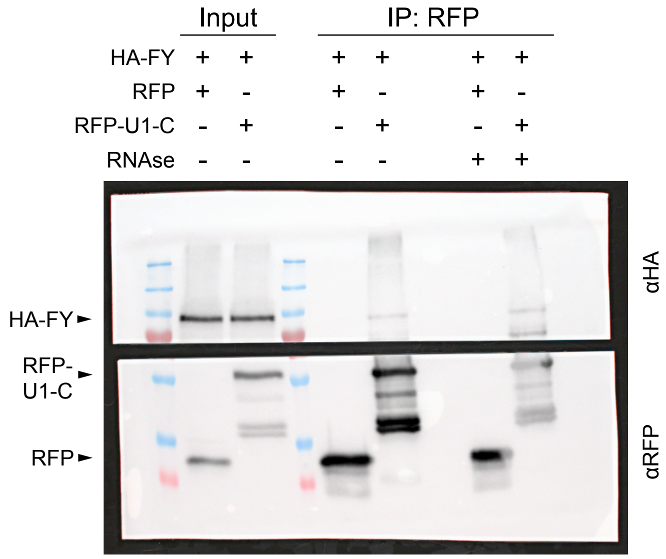

Unprocessed blots for Figure 2F

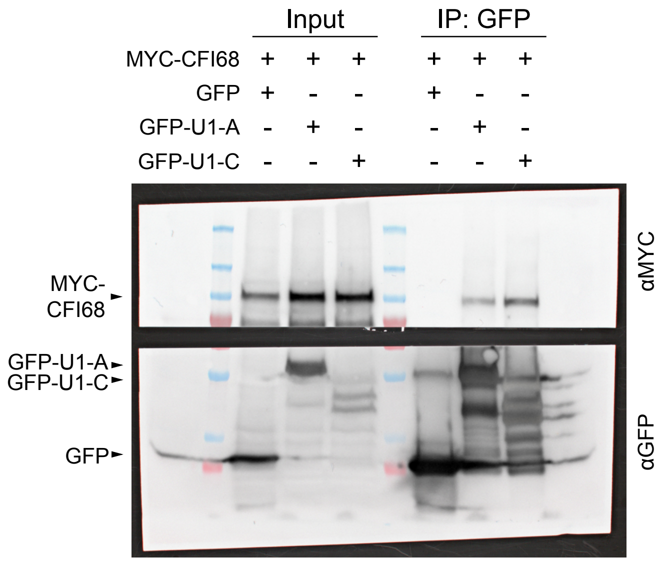

Supplement: Supplementary file 10 — Unprocessed western blots. [file 41477_2024_1796_MOESM10_ESM.pdf]
