# Supplementary figures and images for: The Arabidopsis U1 snRNP regulates mRNA 3′-end processing
Source: Nat Plants. 2024 Sep 23;10(10):1514–31. doi: 10.1038/s41477-024-01796-8 (PMC11489095; doi:10.1038/s41477-024-01796-8)

Unprocessed blots for Extended Data Figure 1A

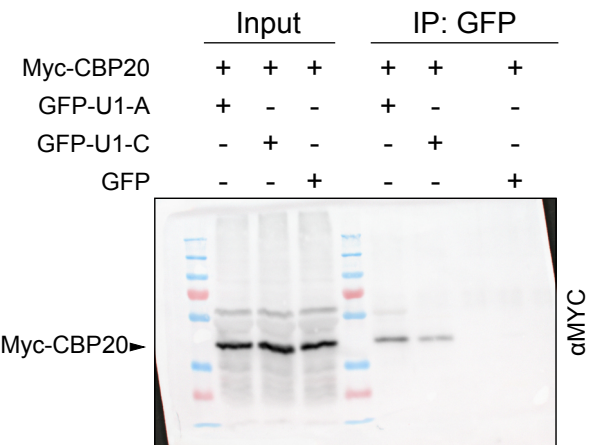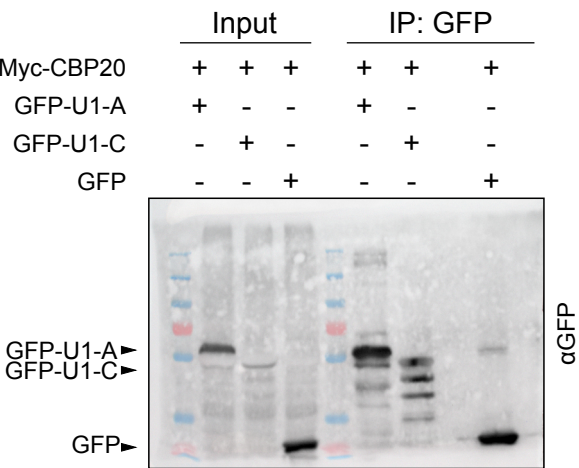

Unprocessed blots for Extended Data Figure 1B

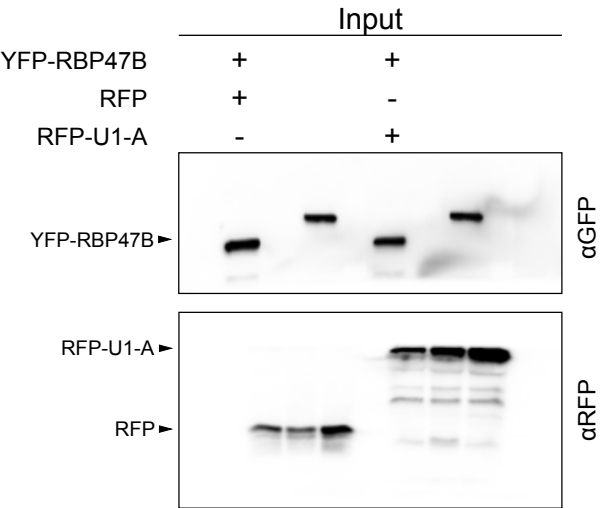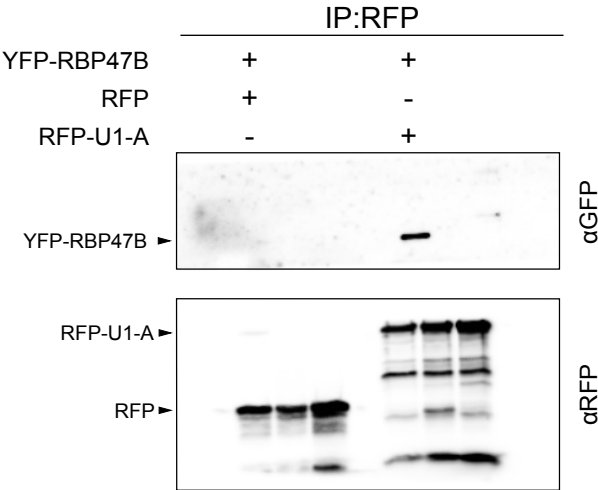

Supplement: Supplementary file 11 — Unprocessed western blots. [file 41477_2024_1796_MOESM11_ESM.pdf]
